# Invasive treatment strategy for older patients with non-ST-elevation acute coronary syndrome: a systematic review and meta-analysis of randomized controlled trials

**DOI:** 10.3389/fcvm.2025.1638932

**Published:** 2025-10-13

**Authors:** Vaibhav Vats, Rai Dilawar Shahjehan, Bavurothu Sharanya Kumar, Keerthi Sanapala, Kartik Mittal, Carlos Andres Barba Herazo, Seema Nabil Nimer, Aishwarya Raparthi, Jasneet K. Arora, Nikhil Kumar Balagoni, Alaa Hamza Hermis, Rawaa M. Mohammad, Huzaifa Ahmad Cheema, Bilawal Nadeem, Muhammad Aslam Khan, Saad Ur Rehman, Muzammil Farhan, Raheel Ahmed

**Affiliations:** ^1^ Department of Internal Medicine, Jacobi Medical Center, Albert Einstein College of Medicine, Bronx, NY, United States; ^2^ Department of Cardiology, University of Texas Medical Branch, Galveston, TX, United States; ^3^ Department of Medicine, Andhra Medical College, Visakhapatnam, India; ^4^ Department of Geriatric Medicine and Longevity, Artemis Hospital, Gurugram, India; ^5^ Department of Emergency, Hospital d’Olot I Comarcal de la Garrotxa, Olot, Spain; ^6^ Department of Medicine, University of Jordan, Amman, Jordan; ^7^ Department of Medicine, All Saints University School of Medicine, Roseau, Dominica; ^8^ Department of Medicine, Osmania Medical College, Hyderabad, India; ^9^ Nursing College, Al-Mustaqbal University, Hillah, Iraq; ^10^ Department of Cardiology, King Edward Medical University, Lahore, Pakistan; ^11^ Department of Internal Medicine, Boston Medical Center, Boston, MA, United States; ^12^ Department of Internal Medicine, Guthrie Robert Packer Hospital, Sayre, PA, United States; ^13^ Division of Cardiovascular Medicine, Department of Medicine, Lahey Hospital and Medical Center, Beth Israel Lahey Health, Burlington, MA, United States; ^14^ National Heart & Lung Institute, Imperial College London, London, United Kingdom; ^15^ Department of Cardiology, Royal Brompton Hospital, London, United Kingdom

**Keywords:** NSTE-ACS, elderly, NSTEMI, invasive management, meta-analysis

## Abstract

**Background:**

The optimal strategy for managing older patients with non-ST-elevation acute coronary syndrome (NSTE-ACS) is uncertain. We aimed to compare the outcomes of invasive vs. conservative strategies for managing NSTE-ACS in older patients ≥65 years.

**Methods:**

We systematically searched MEDLINE, Embase, CENTRAL, and ClinicalTrials.gov, up to March 2025. We included randomized controlled trials (RCTs) comparing a routine invasive treatment strategy with conservative management alone in patients ≥65 years old with NTE-ACS. We pooled risk ratios (RRs) and hazard ratios (HRs) under a random-effects model.

**Results:**

We included 8 RCTs (3,887 patients). There was no significant difference between invasive and conservative management in the risk of a composite outcome of all-cause mortality or MI (RR 0.91, 95% CI: 0.79, 1.06; HR 0.88, 95% CI: 0.74, 1.05), and all-cause mortality (RR 1.05, 95% CI: 0.93, 1.17; HR 1.03, 95% CI: 0.90, 1.19). Invasive management significantly decreased the risk of MI (RR 0.70, 95% CI: 0.55, 0.89) and revascularization (RR 0.29, 95% CI: 0.21, 0.40). There was no significant difference between the two strategies in the risk of cardiovascular mortality (RR 1.09, 95% CI: 0.87, 1.35) and stroke (RR 0.77; 95% CI: 0.53, 1.12). Invasive management increased the incidence of severe bleeding (RR 1.43; 95% CI: 1.05, 1.94).

**Conclusions:**

An invasive strategy in older patients with NSTE-ACS decreased the risk of MI and the need for revascularization. Future RCTs need longer follow-ups and should be conducted in ethnically diverse populations to enhance generalizability.

**Systematic Review Registration:**

https://www.crd.york.ac.uk/PROSPERO/view/CRD42024629566, PROSPERO CRD42024629566.

## Introduction

Non-ST-elevation acute coronary syndrome (NSTE-ACS) is a significant manifestation of ischemic heart disease. It accounts for one of the most important causes of morbidity and mortality around the world. Adults over 65 years account for over half of all NSTE-ACS patients (1). Cardiovascular diseases, which include NSTE-ACS, contribute to around 18 million deaths each year, and most of them occur in the elderly (2). Frailty and comorbidities in the elderly also enhance the risks and make the management more difficult (3).

The management of NSTE-ACS depends on mortality risk stratification, clinical findings, and the availability of resources. Despite guidelines recommending a similar approach for the management of NSTE-ACS across all age groups (4, 5), evidence supporting routine invasive interventions remains ambiguous for elderly populations. Randomized controlled trials (RCTs) investigating an invasive treatment strategy in elderly patients have produced conflicting results (6, 7); however, they were marred by small sample sizes and brief follow-up durations. Prior systematic reviews and meta-analyses on this topic have also been limited by heterogeneity in inclusion criteria, age threshold variations, and a paucity of frail elderly patients included (8). A recent meta-analysis of 2,429 patients found that an invasive strategy for older adults reduced the incidence of a composite of death and myocardial infarction (MI), and MI compared with a conservative approach (9). In contrast, another recent meta-analysis showed that routine invasive management significantly reduced the risk of repeat MI and revascularization; however, this did not translate into consistent reductions in all-cause mortality or cardiovascular death (10). Recently, SENIOR-RITA, the largest trial on this topic to date, with 1,518 patients, was published (11), necessitating a re-evaluation of the current evidence base to resolve inconsistencies between prior analyses and provide new insights into this topic. This systematic review and meta-analysis aims to compare the outcomes of invasive vs. conservative strategies for managing NSTE-ACS in older patients ≥65 years old.

## Methods

This systematic review and meta-analysis were performed and reported following the guidelines established by the Cochrane Collaboration Handbook for Systematic Reviews of Interventions and the Preferred Reporting Items for Systematic Reviews and Meta-Analysis (PRISMA) statement (12, 13). The review was registered in the International Prospective Register of Systematic Reviews (PROSPERO: CRD42024629566).

### Eligibility criteria

Inclusion in this meta-analysis was restricted to studies that met all the following eligibility criteria: (1) RCTs; (2) adult patients ≥65 years old with NTE-ACS; and (3) routine invasive treatment strategy compared to conservative management alone. In addition, studies were included only if they reported any of the outcomes of interest. We excluded single-armed and observational studies.

### Information sources and search strategy

A systematic search was conducted across the Cochrane Central Register of Controlled Trials (CENTRAL, via The Cochrane Library), MEDLINE (via PubMed), Embase, ClinicalTrials.gov, and grey literature sources, including ProQuest Dissertations and Theses Global (PQDT), from inception to March 2025. The search terms used included “adult,” “non-ST elevation acute coronary syndrome,” “NTE-ACS,” “cardiac catheterization,” and “cardiac angiography.” The detailed search strategy is provided in Supplementary Table 1. Additionally, the references of all included studies and prior systematic reviews and meta-analyses were manually reviewed to identify any further relevant studies.

### Selection process

The results from all online literature searches were uploaded into Rayyan Systems, Inc., a software tool designed for article selection. Duplicate articles were removed, after which two authors independently screened the titles and abstracts. The remaining articles were then subjected to a comprehensive full-text review by the same authors. Any disagreements between the two were resolved through discussion with a third author.

### Data collection process and data items

Upon completing the study selection process, two authors independently input the data into a pre-designed Excel spreadsheet to ensure consistency in data collection. Key information was extracted, including patient characteristics such as age, population, percentage of males, history of MI, prior percutaneous coronary intervention (PCI), and prior coronary artery bypass grafting (CABG), study characteristics (country, sample size, and study duration), and outcome variables. The primary outcomes assessed were a primary composite endpoint of all-cause mortality or MI, all-cause mortality, and MI. Secondary outcomes included cardiovascular mortality, stroke, need for revascularization, and severe bleeding.

### Risk of bias assessment

The potential for bias in randomized studies was assessed using the Cochrane Risk of Bias version 2.0 tool (RoB 2.0) (14). This tool evaluates five key domains of potential bias: (1) bias from the randomization process, (2) bias from deviations in intended interventions, (3) bias from missing outcome data, (4) bias in outcome measurement, and (5) bias related to the selection of reported results. Two authors independently conducted the quality assessment of each included study. Any discrepancies were resolved by a third author acting as an arbiter.

### Strategy for data synthesis

We used Review Manager 5.4 software from the Cochrane Collaboration to conduct the meta-analysis. We used a random-effects model to calculate the risk ratio (RR) and 95% confidence interval (CIs) for each outcome. Additionally, wherever enough data were available, we pooled each outcome using the hazard ratio (HR) as the effect measure. We used the Chi^2^ test and the *I*^2^ statistic to assess heterogeneity, considering a *P*-value < 0.10 as statistically significant for the Chi^2^ test.

We performed a subgroup analysis by stratifying studies into two groups: those including NSTEMI patients only and those including all NSTE-ACS patients. We also conducted a sensitivity analysis by excluding studies enrolling patients younger than 70 years old. A *post hoc* sensitivity analysis was performed by only pooling studies reporting major bleeding. We also added a *post hoc* leave-one-out sensitivity analysis for our primary outcomes. Additionally, we conducted meta-regression analyses for our primary outcomes with mean age, male percentage, and percentage of patients with prior MI as the moderators. However, the results of meta-regression should be interpreted with caution, as it is not recommended to be used when the number of studies is less than 10 due to potentially unreliable estimates.

### Certainty of evidence assessment

We assessed the certainty of evidence using the Grading of Recommendations Assessment, Development and Evaluation (GRADE) approach, which considers risk of bias, inconsistency, indirectness, and imprecision (15). Imprecision was judged by evaluating whether the total sample size met the optimal information size (OIS) and whether the CIs intervals crossed thresholds of clinical decision-making.

## Results

**Figure 1:**
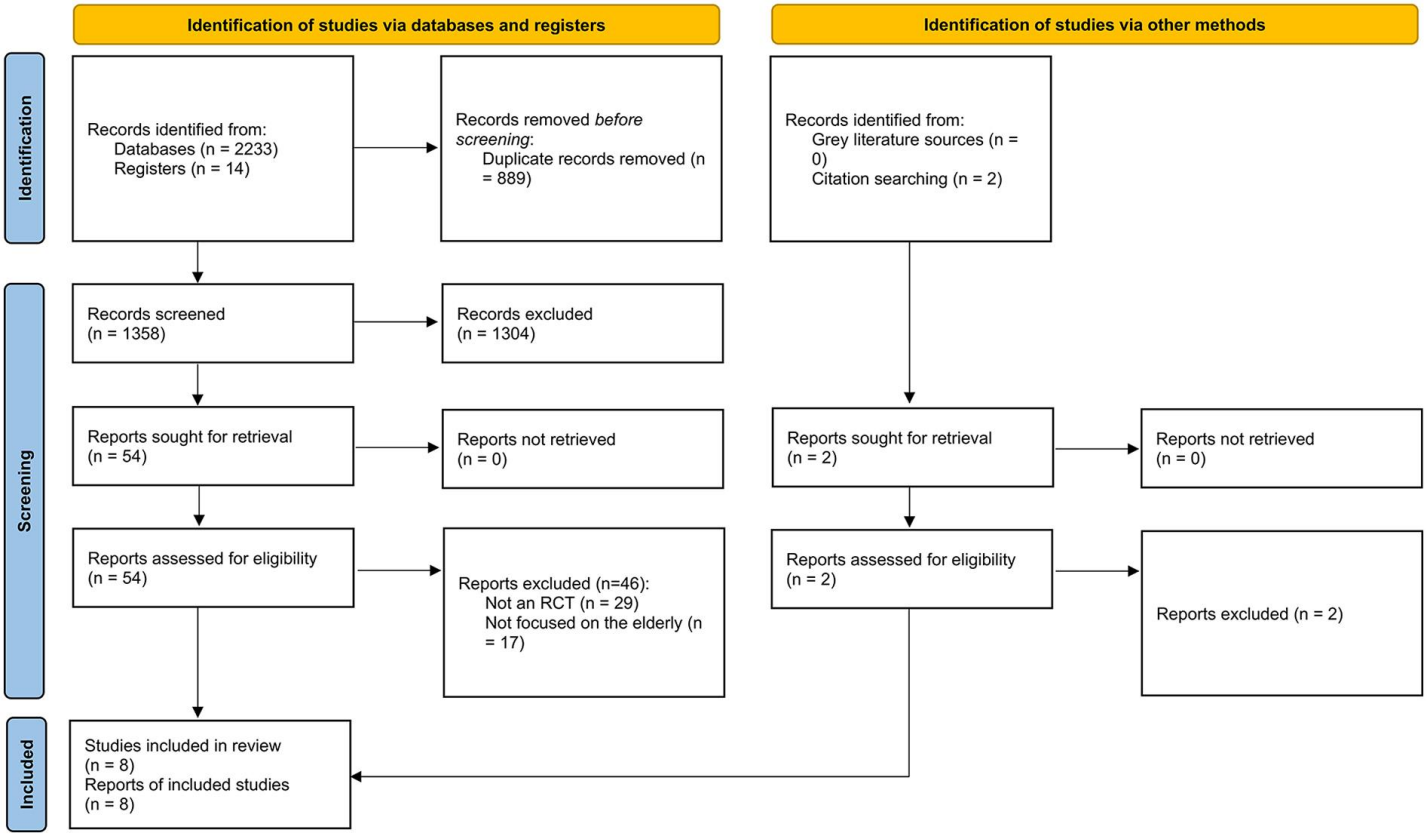
PRISMA 2020 flowchart.

We included 8 RCTs (3,887 patients) in our meta-analysis (Figure 1) (6, 7, 11, 16–20). The trials were conducted in the US and various European countries. Three studies enrolled patients with NSTE-ACS, while the remaining five enrolled patients with NSTEMI only. One study included adults ≥65 years old, two studies ≥70 years old, two studies ≥75 years old, and three studies ≥80 years old. The mean age in all studies but one ranged from 81 to 86. The detailed characteristics of the included trials are presented in Table 1; Supplementary Table 2.

**Table 1:** Characteristics of included studies.

Study ID	trial name	Location	Population	No. of patients	Age (years)	Male (%)	Frail (%)	Prior MI (%)	Previous PCI (%)	Previous CABG (%)	Median no. of days from randomization to angiography	Crossover from conservative to invasive group (%)	Follow-up time
Bach et al. (2004) (16)	TACTICS–TIMI 18	United States	≥65 years with NSTEMI	962 (491 vs 471)	72.9 ± 5.6	59.5	NA	38.3	NA	NA	NA	51.8	Day 30 and at 6 months
de Belder et al. (2021) (17)	RINCAL	UK	≥80 with NSTEMI	251 (125 vs 126)	84.8 (80−95) vs 85.2 (80–95)	51.6 vs 54	NA	26.8 vs 28.5	17.2 vs 12.9	9.7 vs 8.1	2	8.7	Median 369 days
Hirlekar et al. (2020) (7)	80 + study	Sweden	≥80 years with NSTE-ACS	186 (93 vs 93)	84 (81–90) vs 84 (81–89)	50.5 vs 59.1	21 vs 13.4	31.9 vs 37.6	16.3 vs 17.2	20.4 vs 15.1	NA	4.3	1 year
Kunadian (2024) et al. (11)	SENIOR-RITA	UK	≥75 years with NSTEMI	1518 (753 vs 765)	82.5 ± 4.7 vs 82.2 ± 4.7	55.2 vs 55.3	32.3 vs 32.6	32.8 vs 29.7	21.7 vs 18.2	13.4 vs 10.5	3	0	Median 4.1 years
Sanchis et al. (2016) (19)	MOSCA	Spain	≥70 years with NSTEMI	106 (52 vs 54)	81 ± 5 vs 83 ± 6	56 vs 50	NA	46 vs 43	23 vs 17	19 vs 7	NA	20	Median 1.9 years
Sanchis et al. (2023) (18)	MOSCA-FRAIL	Spain	>70 years with NSTEMI	167 (84 vs 83)	86 ± 5 vs 85 ± 5	38 vs 57	73 vs 76	23 vs 39	23 vs 40	6 vs 13	NA	11	1 year
Savonitto et al. (2012) (20)	The Italian Elderly ACS Study	Italy	≥75 years with NSTE-ACS	313 (154 vs 159)	81.8 ± 4.4 vs 81.8 ± 4.7	49 vs 51	NA	28 vs 34	11 vs 20	11 vs 7.6	1	29	1 year
Tegn et al. (2016) (6)	After Eighty	Norway	≥80 years with NSTE-ACS	457 (229 vs 228)	84.7 (80–93) vs 84.9 (80–94)	55 vs 44	NA	47 vs 39	24 vs 20	19 vs 14	1.4	0	Median 1.53 years

### Risk of bias

Four studies had a low risk of bias (7, 11, 17, 20), while the remaining four had some concerns of bias arising in the domains of the randomization process and deviations from intended interventions (6, 16, 18, 19) (Supplementary Figure 1).

## Results of the meta-analysis

### Primary outcomes

**Figure 2:**
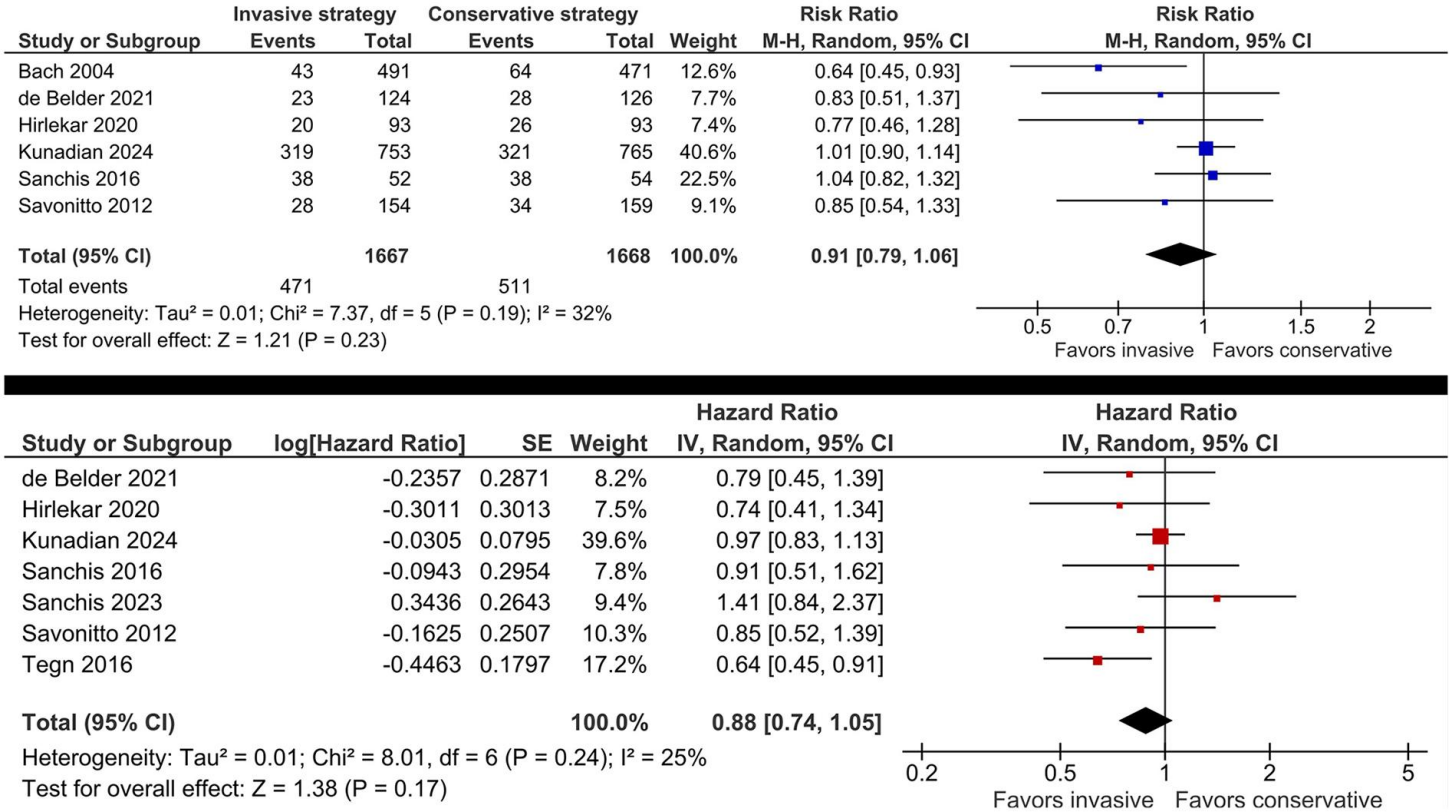
Effect of invasive versus conservative management on the risk of the primary composite outcome of all-cause mortality or MI in older patients with NSTE-ACS.

There was no significant difference between invasive management and conservative management for older adults with NSTE-ACS in the risk of the primary composite outcome of all-cause mortality or MI (RR 0.91, 95% CI: 0.79, 1.06, *I*^2^ = 32%; HR 0.88, 95% CI: 0.74, 1.05, *I*^2^ = 25%; Figure 2). However, when stratified according to trial population, there was a decreased hazard of all-cause mortality or MI with invasive management in patients with NSTE-ACS (HR 0.71, 95% CI: 0.55, 0.92) but not in trials enrolling NSTEMI patients only (HR 0.98, 95% CI: 0.85, 1.13; *P_interaction_* = 0.03; Supplementary Figure 2). Excluding the trial enrolling patients <70 years (16) decreased the heterogeneity (*I*^2^ = 0%); the results remained non-significant.

**Figure 3:**
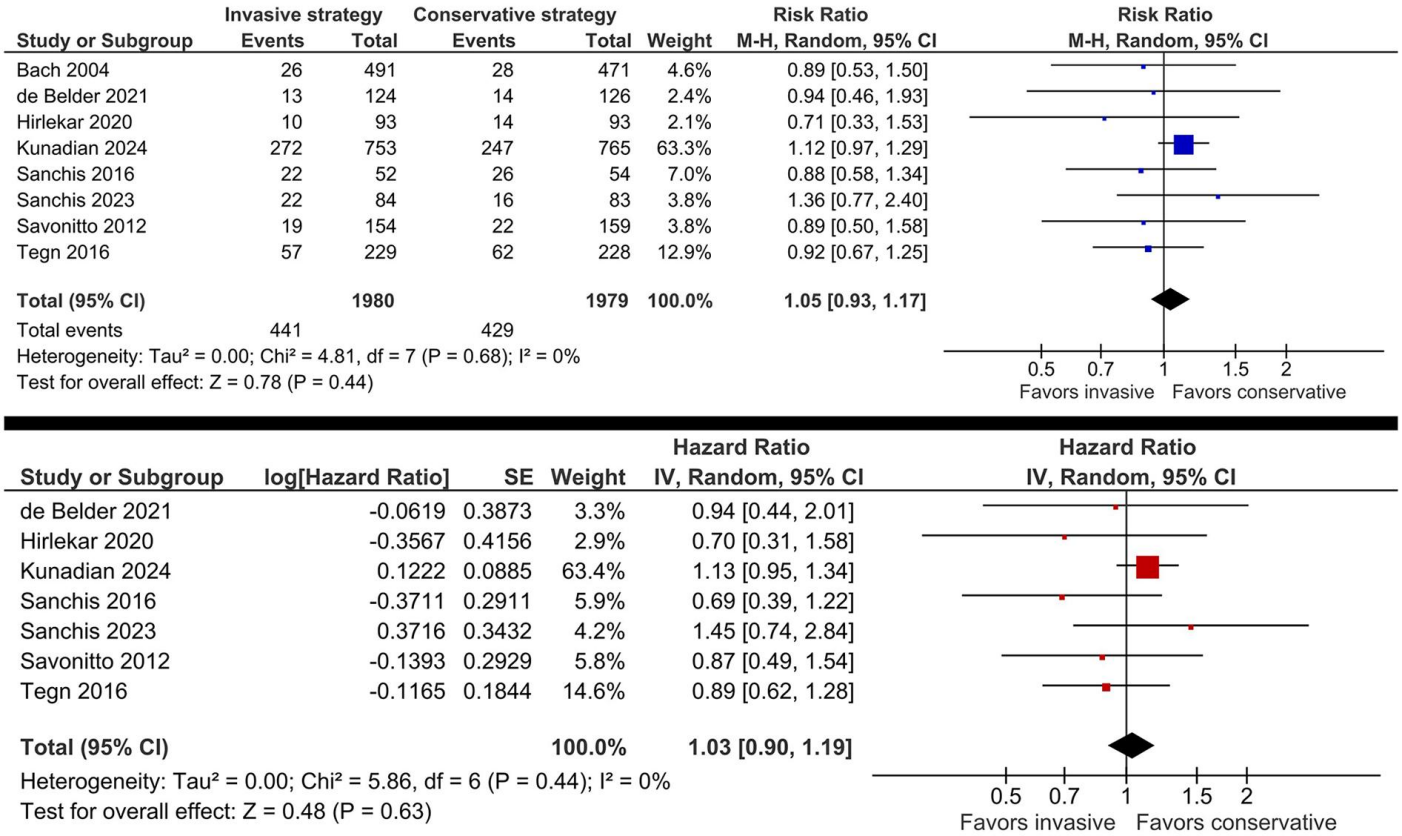
Effect of invasive versus conservative management on the risk of all-cause mortality in older patients with NSTE-ACS.

There was no significant difference between the two strategies in the risk of all-cause mortality (RR 1.05, 95% CI: 0.93, 1.17, *I*^2^ = 0%; HR 1.03, 95% CI: 0.90, 1.19, *I*^2^ = 0%; Figure 3). The results of the subgroup analysis according to the trial population were consistent with the primary analysis (Supplementary Figure 3). Excluding the trial enrolling patients <70 years (16) did not affect the results.

**Figure 4:**
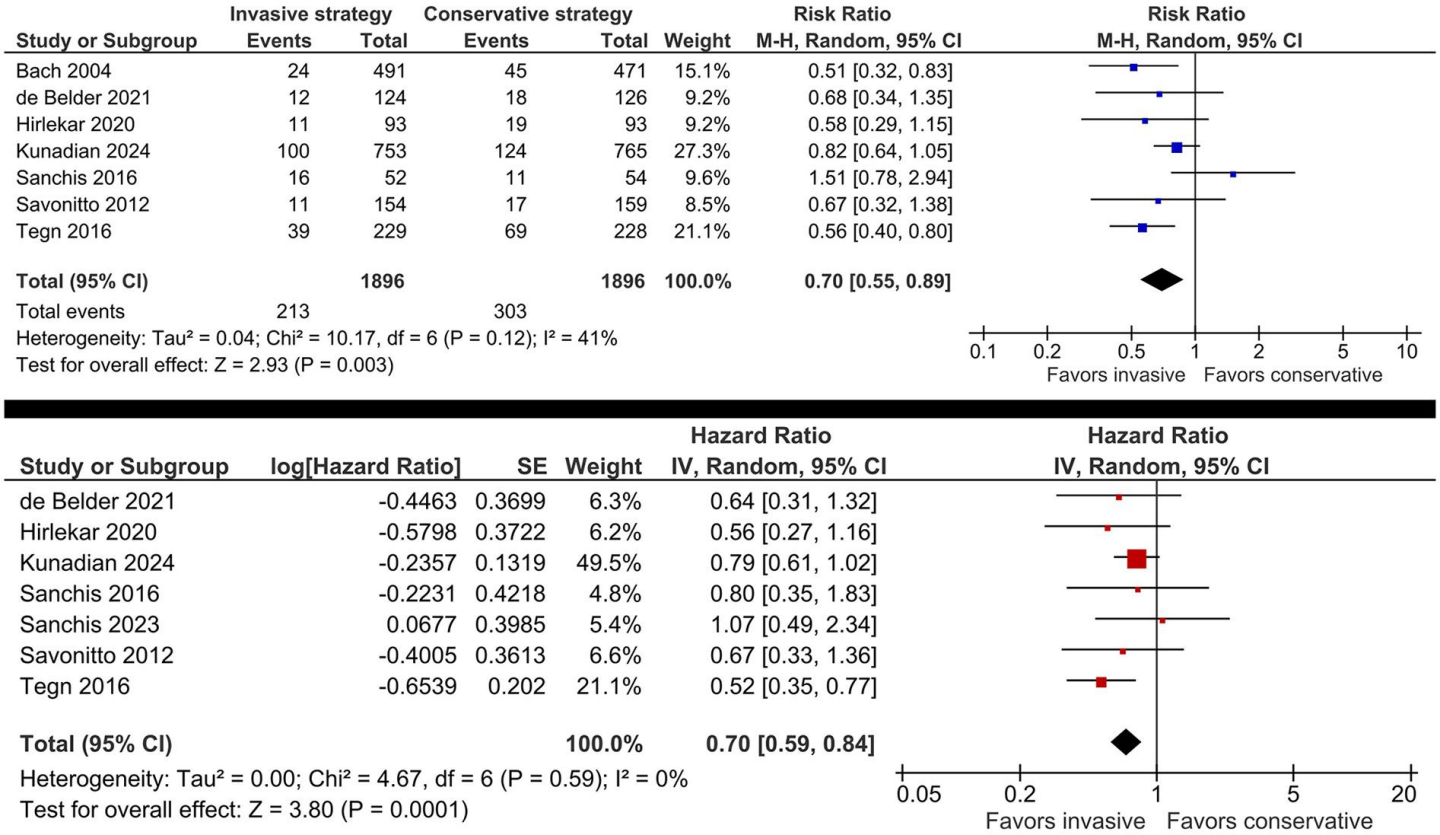
Effect of invasive versus conservative management on the risk of MI in older patients with NSTE-ACS.

Invasive management significantly decreased the risk of MI (RR 0.70, 95% CI: 0.55, 0.89, *I*^2^ = 41%; Figure 4) and increased the time to MI (HR 0.70, 95% CI: 0.59, 0.84, *I*^2^ = 0%; Figure 4). The benefit was consistent across trials enrolling patients with NSTE-ACS and trials including patients with NSTEMI only (Supplementary Figure 4). Sensitivity analysis by excluding the trial with patients <70 years (16) did not impact the results.

### Secondary outcomes

There was no significant difference between the two strategies regarding cardiovascular mortality (RR 1.09, 95% CI: 0.87, 1.35, *I*^2^ = 0%; HR 1.10, 95% CI: 0.86, 1.41, *I*^2^ = 0%; Supplementary Figure 5). An invasive management strategy decreased the risk of revascularization (RR 0.29, 95% CI: 0.21, 0.40, *I*^2^ = 0%; HR 0.28, 95% CI: 0.20, 0.39, *I*^2^ = 0%; Supplementary Figure 6). There were no significant differences between the two groups in the risk of stroke (RR 0.77; 95% CI: 0.53, 1.12, *I*^2^ = 0%; HR 0.78, 95% CI: 0.52, 1.16, *I*^2^ = 0%; Supplementary Figure 7). There was a significant increase in the incidence of severe bleeding in the invasive management group (RR 1.43; 95% CI: 1.05, 1.94, *I*^2^ = 13%; Supplementary Figure 8). When focusing on studies reporting major bleeding, the increased risk of bleeding with the invasive strategy persisted (RR 1.71; 95% CI: 1.15, 2.54, *I*^2^ = 0%; Supplementary Figure 9). Excluding the trial enrolling patients <70 years (16) did not affect the results for any of the secondary outcomes except for severe and major bleeding, which became non-significant (Supplementary Figures 10, 11).

### Leave-one-out sensitivity and meta-regression analyses

Leave-one-out sensitivity analyses indicated that no single study materially influenced the pooled effect estimates for any primary outcome (Supplementary Figures 12–14). Meta-regression analyses using mean age, proportion of male participants, and prior MI showed no significant association with the pooled outcomes (Supplementary Figures 15–S23).

### Certainty of evidence

MI was rated as high certainty, while the rest of the outcomes were downgraded to moderate certainty due to imprecision in the effect estimates. The results of the GRADE assessment are presented in Supplementary Table 3.

## Discussion

In this largest meta-analysis to date, we demonstrate that invasive management in elderly patients, aged 65 and above, presenting with NSTE-ACS does not lead to statistically significant improvement in the primary composite outcome of all-cause mortality or MI compared to a conservative approach. Invasive strategy was found to show a significant decrease in the risk of MI and the need for revascularization. A corresponding decrease was also found in the hazards of MI and revascularization. However, this came at the expense of an increase in bleeding events, although the result was not robust.

This meta-analysis builds on previously conducted RCTs between 2004 and 2024. This study surpasses all previous meta-analyses on this topic in terms of the number of patients and trials included (6, 9, 10, 21), and it analyzed data from 3887 subjects to provide more statistical power and achieve more reliable results. None of the prior meta-analyses showed a mortality benefit; our study validates this finding. Our findings contradict those of the meta-analysis by Rout et al., which showed a decreased risk of the composite end point of death and MI (odds ratio 0.67; *p* < 001) (10). Similarly, in contrast to our results, the meta-analysis by Saraswat et al. showed a reduction in 30-day mortality, 1-year mortality, and events of stroke, and the meta-analysis by Khalil et al. showed no significant findings for the outcomes of MI, revascularization, and bleeding events (9, 21). These differences may be due to the inclusion of observational studies or fewer RCTs in previous analyses. Additionally, we analyzed time-to-event data and calculated HRs for our outcomes to provide a more dynamic measure of risk. Unlike RRs or odds ratios (ORs), which compare overall probabilities, HRs account for when events occur, making them particularly powerful for assessing differences in event timing and progression; this is particularly relevant in elderly patients with NSTE-ACS, where event timing and variable follow-up may influence outcome estimates. We also systematically examined bleeding definitions, vascular access, and antithrombotic regimens across trials, providing context to interpret variability in bleeding risk and highlighting contemporary strategies to mitigate it. Finally, sensitivity analyses excluding specific trials and meta-regression by mean age offer insights into how trial characteristics and age-related heterogeneity influence outcomes. Collectively, these approaches extend prior findings and provide a detailed, clinically relevant synthesis for guiding management decisions in the oldest and most complex patients.

An interesting finding in our subgroup analyses was that invasive management showed a benefit in the primary composite outcome in trials enrolling patients with NSTE-ACS but not in patients with NSTEMI only. However, this needs to be interpreted with caution as subgroup analyses are observational in nature and hence, may be confounded by other uncontrolled variables. Future RCTs should investigate all subtypes of NSTE-ACS to provide more definitive conclusions.

The 2021 guidelines by the American College of Cardiology (ACC)/American Heart Association (AHA)/Society of Coronary Angiography and Interventions (SCAI) and the 2023 guidelines by the European Society of Cardiology (ESC) recommend similar approaches to treat NSTE-ACS, irrespective of age groups (22, 23). The elderly population is usually frail with multiple comorbidities and sometimes cannot tolerate coronary angiography or intervention well. Chronic kidney disease (CKD) is a common comorbidity among elderly patients, and at times, contrast exposure leads to dialysis need (24). It might be suggested that current guidelines on the management approaches to NSTE-ACS be revised based on age groups. Increased bleeding risk seen in the invasive arm is most likely due to mandatory use of dual antiplatelet therapy after percutaneous coronary intervention, which is less well tolerated in the elderly population compared to younger patients (25). However, this result should be interpreted with due caution, as excluding the TACTICS-TIMI 18 trial in a sensitivity analysis rendered the observed increase in bleeding with the invasive strategy non-significant. This highlights the influence of this early study, which had substantial crossover, limited follow-up, and lacked detailed patient-level data, underscoring the need to interpret bleeding outcomes in the context of individual trial characteristics. Elderly patients experience worse outcomes from NSTE-ACS than younger adults, underscoring the importance of personalized risk assessments and a focus on patient-centered care decision-making (26).

Contemporary strategies have been shown to reduce bleeding risk in elderly patients undergoing invasive management for NSTE-ACS. Radial artery access is increasingly preferred over femoral access, as it is consistently associated with lower rates of vascular complications and major bleeding (27). Optimization of antithrombotic therapy, including careful selection and dosing of anticoagulants and antiplatelet agents, can further mitigate bleeding risk (28). Shorter durations of dual antiplatelet therapy, when clinically appropriate, and the routine use of gastroprotective agents such as proton pump inhibitors, particularly in patients at high gastrointestinal bleeding risk, are also recommended (29, 30). Incorporating these strategies may help reconcile the elevated bleeding risk observed in earlier studies with outcomes in contemporary practice, highlighting that individualized management can improve the safety of invasive approaches in older adults.

Although our study provides important insight into the implications of an invasive approach towards the elderly population, it does come with limitations. We used trial-level data instead of patient-level data, which precludes adjustment for individual patient characteristics such as frailty, comorbidities, or extreme age, limiting the applicability of our findings to the highest-risk elderly populations. This study included patients >65 years of age; however, most of the patient population is above >80 years old, which might make results not generalizable to patients in their late 60s and 70s. The median follow-up period for the trials we included is 1 year only, and the long-term effects of invasive therapeutic approaches for the elderly population are still unknown. The lack of access to individual patient-level data and the incomplete reporting of procedural details and antithrombotic regimens by the included randomized trials precluded detailed subgroup analyses of bleeding risk to investigate how these factors could influence it. Moreover, the lack of uniformity and clear reporting of the timing of invasive strategy across all our included trials introduces a chance of unquantified heterogeneity, further compounded by disparities in inclusion criteria among these studies. Finally, several included trials reported crossovers from conservative to invasive arms; however, as per-protocol or instrumental variable analyses were not provided, we were unable to adjust for these crossovers, which may have attenuated observed treatment effects.

Future RCTs may follow patients up to several years, which might show different long-term effects of invasive strategies than we expect in terms of all-cause mortality, myocardial infarction, stroke, major bleeding, and need for repeat revascularization. RCTs must be conducted in countries other than Europe and North America to generalize the results for all ethnic groups. Additional variables should be studied, especially nephropathy, progression to end-stage kidney disease, new onset cardiomyopathy, and drop in ejection fraction.

## Conclusions

In this meta-analysis of elderly patients presenting with NSTE-ACS, we showed that invasive management does not lead to statistically significant improvement in the primary composite outcome of all-cause mortality or MI compared to a conservative approach. Invasive strategy was found to show a significant decrease in the risk of MI and the need for revascularization. While there was an increase in bleeding events with an invasive management approach, this was not robust and largely driven by an early trial with significant limitations. Future RCTs need longer follow-ups and should be conducted in ethnically diverse populations to enhance generalizability.

## Data Availability

The original contributions presented in the study are included in the article/Supplementary Material, further inquiries can be directed to the corresponding authors.
