# Supplementary material for: Invasive treatment strategy for older patients with non-ST-elevation acute coronary syndrome: a systematic review and meta-analysis of randomized controlled trials
Source: Front Cardiovasc Med. 2025 Oct 13;12:1638932. doi: 10.3389/fcvm.2025.1638932 (PMC12554759; doi:10.3389/fcvm.2025.1638932)
Supplement: Supplementary file 1 [file Datasheet1.pdf]

Table S1: Search strategy for online databases.

PubMed/MEDLINE:

(“NSTACS” OR “NSTEMI” OR “NSTE-ACS” OR “NSTEACS” OR “Acute Myocardial Ischemia” OR ((“Non-ST Elevated” OR “Non-ST-Elevated” OR “Non ST Elevated” OR “Non-ST-Elevation” OR “Non ST Elevation” OR “Non-ST Elevation”) AND (“Myocardial Infarction”)) OR “acute coronary syndrome” OR “ACS”) AND (“early invasive” OR "Invasive strategy" OR "invasive approach" OR "coronary angiography" OR "percutaneous coronary intervention" OR “PCI” OR "revascularization" OR "coronary artery bypass grafting" OR “CABG” OR “Coronary Revascularization” OR “Angioplast*” OR “Coronary Intervention” OR “Coronary Atherectom*”) AND ("Conservative strategy" OR "conservative approach" OR "guideline-directed medical therapy" OR “GDMT” OR "pharmacological therapy" OR “standard care” OR “medical therap*” OR “OMT” OR “medical treatment” OR “medical management” OR “conservative management” OR “conservative treatment” OR “conservative therap*”) AND (“Stroke” OR "heart failure" OR "Mortality" OR "cardiovascular death" OR "myocardial infarction" OR “MACE” OR "Major Adverse Cardiovascular Events" OR "re-infarction" OR "major bleeding" OR "complications" OR "hospital stay" OR "readmission" OR "quality of life") AND ("Elder*" OR "older adults" OR "aged 75" OR "geriatric" OR “80 and over” OR “aged >” OR “Aged ≥” OR “frail” OR “octogenarians” OR "septuagenarians" OR "nonagenarians" OR “advanced age” OR “advanced-aged” OR “senior citizens” OR “aged population” OR "older population" OR "older individuals" OR "aged 65" OR "aged 85”)

Embase:

(“NSTACS” OR “NSTEMI” OR “NSTE-ACS” OR “NSTEACS” OR “Acute Myocardial Ischemia” OR ((“Non-ST Elevated” OR “Non-ST-Elevated” OR “Non ST Elevated” OR “Non-ST-Elevation” OR “Non ST Elevation” OR “Non-ST Elevation”) AND (“Myocardial Infarction”)) OR “acute coronary syndrome” OR “ACS”):TI,AB,KW AND (“early invasive” OR "Invasive strategy" OR "invasive approach" OR "coronary angiography" OR "percutaneous coronary intervention" OR “PCI” OR "revascularization" OR "coronary artery bypass grafting" OR “CABG” OR “Coronary Revascularization” OR “Angioplast*” OR “Coronary Intervention” OR “Coronary Atherectom*”):TI,AB,KW AND ("Conservative strategy" OR "conservative approach" OR "guideline-directed medical therapy" OR “GDMT” OR "pharmacological therapy" OR “standard care” OR “medical therap*” OR “OMT” OR “medical treatment” OR “medical management” OR “conservative management” OR “conservative treatment” OR “conservative therap*”):TI,AB,KW AND (“Stroke” OR "heart failure" OR "Mortality" OR "cardiovascular death" OR "myocardial infarction" OR “MACE” OR "Major Adverse Cardiovascular Events" OR "re-infarction" OR "major bleeding" OR "complications" OR "hospital stay" OR "readmission" OR "quality of life") AND ("Elder*" OR "older adults" OR "aged 75" OR "geriatric" OR “80 and over” OR “aged >” OR “Aged ≥” OR “frail” OR “octogenarians” OR "septuagenarians" OR "nonagenarians" OR “advanced age” OR “advanced-aged” OR “senior citizens” OR “aged population” OR "older population" OR "older individuals" OR "aged 65" OR "aged 85”)

Table S2: Bleeding definitions adopted by the trials and associated variables.

Study Name	Vascular Access	Antithrombotic Regimen	Antiplatelet Regimen	Definition of Bleeding
Bach 2004	Unspecified	IV unfractionated heparin to target aPTT 60–85	Aspirin 325 mg and Tirofiban loading, followed by maintenance ×48 h or until PCI	Decrease in hemoglobin ≥5 g/dL, bleeding requiring ≥2 units transfusion, corrective surgery, intracranial/retroperitoneal hemorrhage, or cardiac tamponade.
de Belder 2021	Predominantly radial (83.3%)	LMWH or Factor Xa inhibitor	Aspirin + P2Y12 inhibitor	Bleeding Academic Research Consortium (BARC) type 3B or above
Hirlekar 2020	Unspecified	As per ESC 2015 guidelines (unspecified)	As per ESC 2015 guidelines (unspecified)	Intracranial bleeding, hemoglobin drop >5 g/dL, or bleeding requiring surgery
Kunadian 2024	Radial access (89.3%)	Apixaban, Rivaroxaban, Warfarin, Edoxaban, Dabigatran	Aspirin + P2Y12 antagonist	BARC type 2 or greater
Sanchis 2016	Radial access (91%)	Anticoagulation unspecified	Aspirin + Clopidogrel	TIMI ≥2 minor bleeding during admission or requiring repeat hospitalization
Sanchis 2023	Radial access (84%)	NOACs (unspecified)	Aspirin + Clopidogrel (69%) or Ticagrelor (10%)	Bleeding requiring rehospitalization
Savonitto 2012	Unspecified	Unfractionated Heparin to target aPTT 50–70 or Enoxaparin	Aspirin + Clopidogrel	Intracranial/retroperitoneal bleeding or clinically overt bleeding with hemoglobin drop ≥5 g/dL or hematocrit decrease ≥15%
Tegn 2016	Radial access (90%)	Optimal medical therapy (unspecified)	Optimal medical therapy (unspecified)	TIMI major bleeding: intracranial or fatal bleeding, or clinically overt hemorrhage with hemoglobin drop ≥5 g/dL

Table S3: GRADE (Grading of Recommendations Assessment, Development and Evaluation) assessment.

Outcome	No. of participants (studies)	Effect estimates (95% CI)	Risk of bias	Inconsistency	Indirectness	Imprecision	Quality of Evidence (GRADE)
Primary composite endpoint	3,335 (6)	RR 0.91 (0.79–1.06)	Not serious	Not serious (I^2=32%)	Not serious	Serious	**Moderate** ⊕⊕⊕⊖
All-cause mortality	3,960 (8)	RR 1.05 (0.93–1.17)	Not serious	Not serious (I^2=0%)	Not serious	Serious	**Moderate** ⊕⊕⊕⊖
Myocardial infarction	3,792 (7)	RR 0.70 (0.55–0.89)	Not serious	Not serious (I^2=41%)	Not serious	Not serious	**High** ⊕⊕⊕⊕
Cardiovascular mortality	2,081 (3)	RR 1.09 (0.87–1.35)	Not serious	Not serious (I^2=0%)	Not serious	Serious	**Moderate** ⊕⊕⊕⊖
Revascularization	2,830 (7)	RR 0.29 (0.21–0.40)	Not serious	Not serious (I^2=0%)	Not serious	Serious	**Moderate** ⊕⊕⊕⊖
Stroke	3,214 (6)	RR 0.77 (0.53–1.12)	Not serious	Not serious (I^2=0%)	Not serious	Serious	**Moderate** ⊕⊕⊕⊖
Severe bleeding	3,960 (8)	RR 1.43 (1.05–1.94)	Not serious	Not serious (I^2=13%)	Not serious	Serious	**Moderate** ⊕⊕⊕⊖

Supplementary Figure S1. Quality assessment of included trials.

Study ID	D1	D2	D3	D4	D5	Overall
Bach 2004	+	+	+	+	+	+
de Belder 2021	+	!	+	+	+	!
Hirlekar 2020	!	!	+	+	+	!
Kunadian 2024	+	!	+	+	+	!
Sanchis 2016	+	+	+	+	+	+
Sanchis 2023	+	+	+	+	+	+
Savonitto 2012	+	!	+	+	+	!
Tegn 2016	+	+	+	+	+	+

+ Low risk
! Some concerns
- High risk

D1 Randomisation process
D2 Deviations from the intended interventions
D3 Missing outcome data
D4 Measurement of the outcome
D5 Selection of the reported result

Supplementary Figure S2. Subgroup analysis for the primary composite outcome of all-cause mortality or MI.

Study or Subgroup	Invasive strategy Events	Invasive strategy Total	Conservative strategy Events	Conservative strategy Total	Weight	Risk Ratio M-H, Random, 95% CI
**2.1.1 NSTEMI**						
Bach 2004	43	491	64	471	12.6%	0.64 [0.45, 0.93]
de Belder 2021	23	124	28	126	7.7%	0.83 [0.51, 1.37]
Kunadian 2024	319	753	321	765	40.6%	1.01 [0.90, 1.14]
Sanchis 2016	38	52	38	54	22.5%	1.04 [0.82, 1.32]
**Subtotal (95% CI)**		**1420**		**1416**	**83.5%**	**0.92 [0.76, 1.11]**
Total events	423		451			
Heterogeneity: Tau² = 0.02; Chi² = 6.20, df = 3 (P = 0.10); I² = 52%						
Test for overall effect: Z = 0.86 (P = 0.39)						
**2.1.2 NSTE-ACS**						
Hirlekar 2020	20	93	26	93	7.4%	0.77 [0.46, 1.28]
Savonitto 2012	28	154	34	159	9.1%	0.85 [0.54, 1.33]
**Subtotal (95% CI)**		**247**		**252**	**16.5%**	**0.81 [0.58, 1.14]**
Total events	48		60			
Heterogeneity: Tau² = 0.00; Chi² = 0.08, df = 1 (P = 0.77); I² = 0%						
Test for overall effect: Z = 1.20 (P = 0.23)						
**Total (95% CI)**		**1667**		**1668**	**100.0%**	**0.91 [0.79, 1.06]**
Total events	471		511			
Heterogeneity: Tau² = 0.01; Chi² = 7.37, df = 5 (P = 0.19); I² = 32%						
Test for overall effect: Z = 1.21 (P = 0.23)						
Test for subgroup differences: Chi² = 0.39, df = 1 (P = 0.53), I² = 0%						

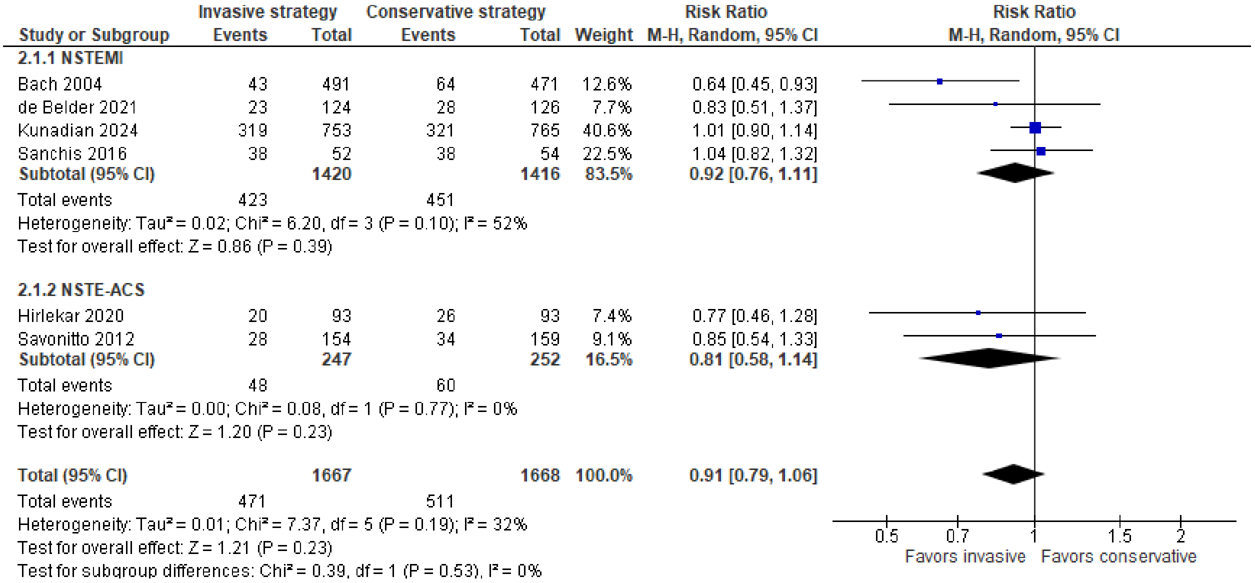

Study or Subgroup	log[Hazard Ratio]	SE	Weight	Hazard Ratio IV, Random, 95% CI
**2.2.1 NSTEMI**				
de Belder 2021	-0.2357	0.2871	8.2%	0.79 [0.45, 1.39]
Kunadian 2024	-0.0305	0.0795	39.6%	0.97 [0.83, 1.13]
Sanchis 2016	-0.0943	0.2954	7.8%	0.91 [0.51, 1.62]
Sanchis 2023	0.3436	0.2643	9.4%	1.41 [0.84, 2.37]
**Subtotal (95% CI)**			**65.0%**	**0.98 [0.85, 1.13]**
Heterogeneity: Tau² = 0.00; Chi² = 2.54, df = 3 (P = 0.47); I² = 0%				
Test for overall effect: Z = 0.27 (P = 0.78)				
**2.2.2 NSTE-ACS**				
Hirlekar 2020	-0.3011	0.3013	7.5%	0.74 [0.41, 1.34]
Savonitto 2012	-0.1625	0.2507	10.3%	0.85 [0.52, 1.39]
Tegn 2016	-0.4463	0.1797	17.2%	0.64 [0.45, 0.91]
**Subtotal (95% CI)**			**35.0%**	**0.71 [0.55, 0.92]**
Heterogeneity: Tau² = 0.00; Chi² = 0.87, df = 2 (P = 0.65); I² = 0%				
Test for overall effect: Z = 2.59 (P = 0.010)				
**Total (95% CI)**			**100.0%**	**0.88 [0.74, 1.05]**
Heterogeneity: Tau² = 0.01; Chi² = 8.01, df = 6 (P = 0.24); I² = 25%				
Test for overall effect: Z = 1.38 (P = 0.17)				
Test for subgroup differences: Chi² = 4.61, df = 1 (P = 0.03), I² = 78.3%				

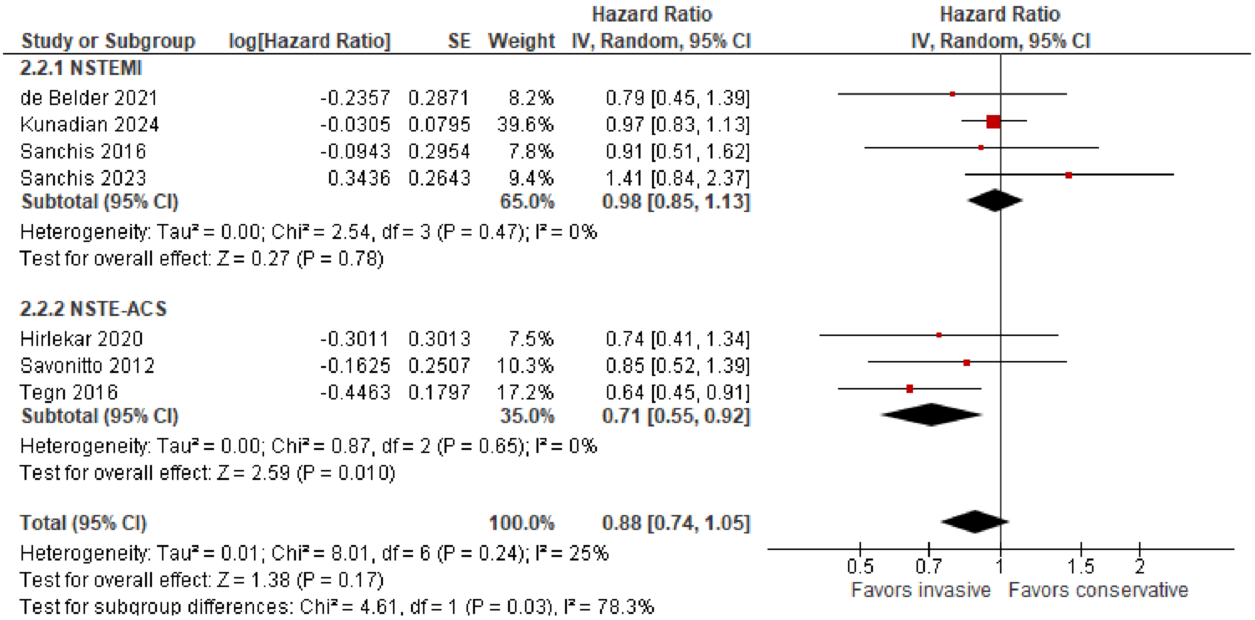

Supplementary Figure S3. Subgroup analysis for all-cause mortality.

Study or Subgroup	Invasive strategy Events	Invasive strategy Total	Conservative strategy Events	Conservative strategy Total	Weight	Risk Ratio M-H, Random, 95% CI
**2.3.1 NSTEMI**						
Bach 2004	26	491	28	471	4.6%	0.89 [0.53, 1.50]
de Belder 2021	13	124	14	126	2.4%	0.94 [0.46, 1.93]
Kunadian 2024	272	753	247	765	63.3%	1.12 [0.97, 1.29]
Sanchis 2016	22	52	26	54	7.0%	0.88 [0.58, 1.34]
Sanchis 2023	22	84	16	83	3.8%	1.36 [0.77, 2.40]
**Subtotal (95% CI)**		**1504**		**1499**	**81.1%**	**1.09 [0.96, 1.23]**
Total events	355		331			
Heterogeneity: Tau² = 0.00; Chi² = 2.45, df = 4 (P = 0.65); I² = 0%						
Test for overall effect: Z = 1.31 (P = 0.19)						
**2.3.2 NSTE-ACS**						
Hirlekar 2020	10	93	14	93	2.1%	0.71 [0.33, 1.53]
Savonitto 2012	19	154	22	159	3.8%	0.89 [0.50, 1.58]
Tegn 2016	57	229	62	228	12.9%	0.92 [0.67, 1.25]
**Subtotal (95% CI)**		**476**		**480**	**18.9%**	**0.89 [0.69, 1.14]**
Total events	86		98			
Heterogeneity: Tau² = 0.00; Chi² = 0.35, df = 2 (P = 0.84); I² = 0%						
Test for overall effect: Z = 0.93 (P = 0.35)						
**Total (95% CI)**		**1980**		**1979**	**100.0%**	**1.05 [0.93, 1.17]**
Total events	441		429			
Heterogeneity: Tau² = 0.00; Chi² = 4.81, df = 7 (P = 0.68); I² = 0%						
Test for overall effect: Z = 0.78 (P = 0.44)						
Test for subgroup differences: Chi² = 1.99, df = 1 (P = 0.16), I² = 49.7%						

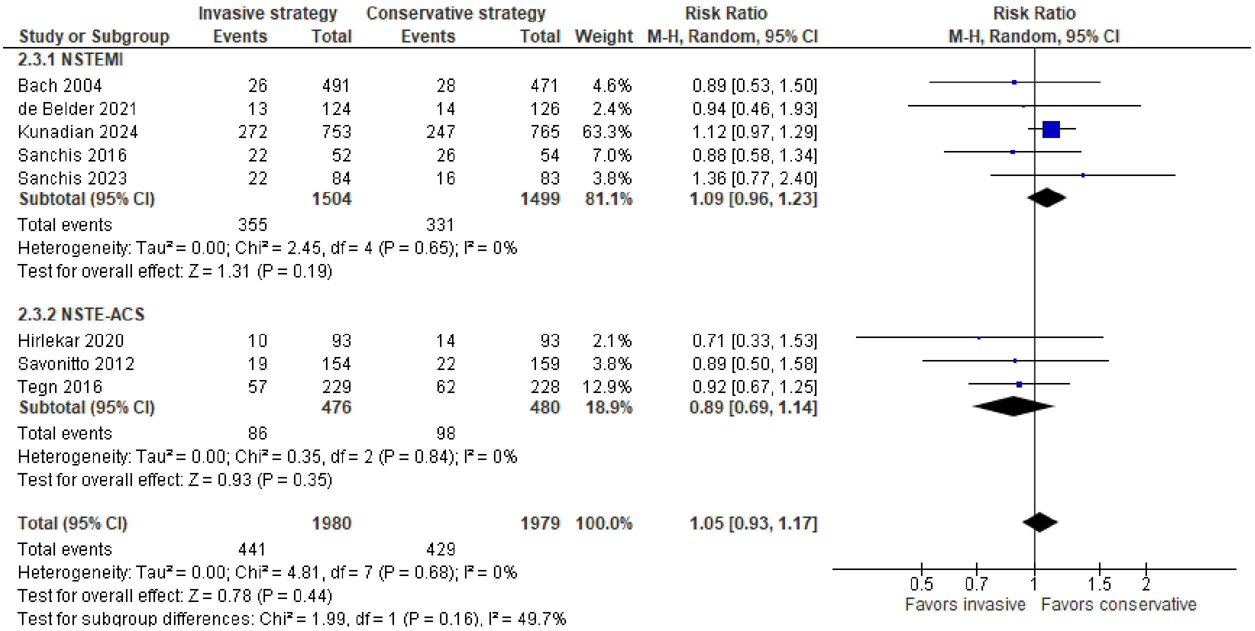

Study or Subgroup	log[Hazard Ratio]	SE	Weight	Hazard Ratio IV, Random, 95% CI
**2.4.1 NSTEMI**				
de Belder 2021	-0.0619	0.3873	3.3%	0.94 [0.44, 2.01]
Kunadian 2024	0.1222	0.0885	63.4%	1.13 [0.95, 1.34]
Sanchis 2016	-0.3711	0.2911	5.9%	0.69 [0.39, 1.22]
Sanchis 2023	0.3716	0.3432	4.2%	1.45 [0.74, 2.84]
**Subtotal (95% CI)**			**76.7%**	**1.07 [0.86, 1.33]**
Heterogeneity: Tau² = 0.01; Chi² = 3.47, df = 3 (P = 0.32); I² = 14%				
Test for overall effect: Z = 0.61 (P = 0.54)				
**2.4.2 NSTE-ACS**				
Hirlekar 2020	-0.3567	0.4156	2.9%	0.70 [0.31, 1.58]
Savonitto 2012	-0.1393	0.2929	5.8%	0.87 [0.49, 1.54]
Tegn 2016	-0.1165	0.1844	14.6%	0.89 [0.62, 1.28]
**Subtotal (95% CI)**			**23.3%**	**0.86 [0.65, 1.14]**
Heterogeneity: Tau² = 0.00; Chi² = 0.28, df = 2 (P = 0.87); I² = 0%				
Test for overall effect: Z = 1.04 (P = 0.30)				
**Total (95% CI)**			**100.0%**	**1.03 [0.90, 1.19]**
Heterogeneity: Tau² = 0.00; Chi² = 5.86, df = 6 (P = 0.44); I² = 0%				
Test for overall effect: Z = 0.48 (P = 0.63)				
Test for subgroup differences: Chi² = 1.43, df = 1 (P = 0.23), I² = 30.3%				

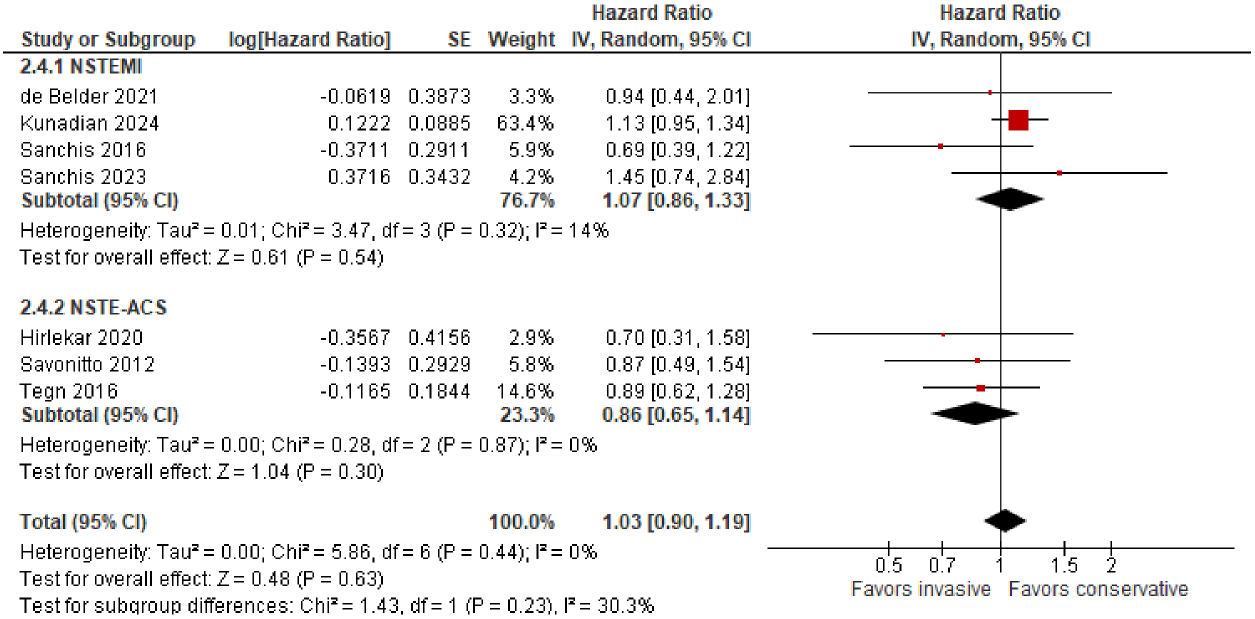

Supplementary Figure S4. Subgroup analysis for MI.

Study or Subgroup	Invasive strategy Events	Invasive strategy Total	Conservative strategy Events	Conservative strategy Total	Weight	Risk Ratio M-H, Random, 95% CI
**2.5.1 NSTEMI**						
Bach 2004	24	491	45	471	15.1%	0.51 [0.32, 0.83]
de Belder 2021	12	124	18	126	9.2%	0.68 [0.34, 1.35]
Kunadian 2024	100	753	124	765	27.3%	0.82 [0.64, 1.05]
Sanchis 2016	16	52	11	54	9.6%	1.51 [0.78, 2.94]
**Subtotal (95% CI)**		**1420**		**1416**	**61.2%**	**0.79 [0.54, 1.14]**
Total events	152		198			
Heterogeneity: $Tau^2 = 0.08$; $Chi^2 = 7.09$, df = 3 (P = 0.07); $I^2 = 58\%$						
Test for overall effect: Z = 1.27 (P = 0.20)						
**2.5.2 NSTE-ACS**						
Hirlekar 2020	11	93	19	93	9.2%	0.58 [0.29, 1.15]
Savonitto 2012	11	154	17	159	8.5%	0.67 [0.32, 1.38]
Tegn 2016	39	229	69	228	21.1%	0.56 [0.40, 0.80]
**Subtotal (95% CI)**		**476**		**480**	**38.8%**	**0.58 [0.44, 0.77]**
Total events	61		105			
Heterogeneity: $Tau^2 = 0.00$; $Chi^2 = 0.18$, df = 2 (P = 0.92); $I^2 = 0\%$						
Test for overall effect: Z = 3.74 (P = 0.0002)						
**Total (95% CI)**		**1896**		**1896**	**100.0%**	**0.70 [0.55, 0.89]**
Total events	213		303			
Heterogeneity: $Tau^2 = 0.04$; $Chi^2 = 10.17$, df = 6 (P = 0.12); $I^2 = 41\%$						
Test for overall effect: Z = 2.93 (P = 0.003)						
Test for subgroup differences: $Chi^2 = 1.59$, df = 1 (P = 0.21), $I^2 = 37.1\%$						

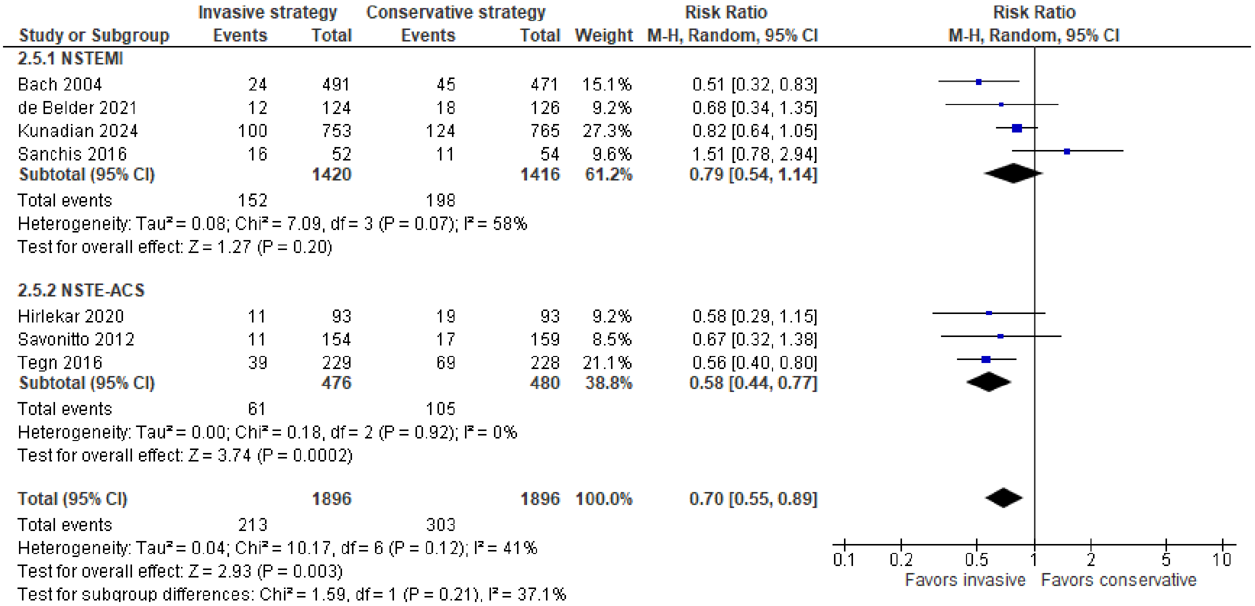

Study or Subgroup	log[Hazard Ratio]	SE	Weight	Hazard Ratio IV, Random, 95% CI
**2.6.1 NSTEMI**				
de Belder 2021	-0.4463	0.3699	6.3%	0.64 [0.31, 1.32]
Kunadian 2024	-0.2357	0.1319	49.5%	0.79 [0.61, 1.02]
Sanchis 2016	-0.2231	0.4218	4.8%	0.80 [0.35, 1.83]
Sanchis 2023	0.0677	0.3985	5.4%	1.07 [0.49, 2.34]
**Subtotal (95% CI)**			**66.1%**	**0.79 [0.64, 0.99]**
Heterogeneity: $Tau^2 = 0.00$; $Chi^2 = 0.90$, df = 3 (P = 0.82); $I^2 = 0\%$				
Test for overall effect: Z = 2.01 (P = 0.04)				
**2.6.2 NSTE-ACS**				
Hirlekar 2020	-0.5798	0.3722	6.2%	0.56 [0.27, 1.16]
Savonitto 2012	-0.4005	0.3613	6.6%	0.67 [0.33, 1.36]
Tegn 2016	-0.6539	0.202	21.1%	0.52 [0.35, 0.77]
**Subtotal (95% CI)**			**33.9%**	**0.55 [0.41, 0.76]**
Heterogeneity: $Tau^2 = 0.00$; $Chi^2 = 0.38$, df = 2 (P = 0.83); $I^2 = 0\%$				
Test for overall effect: Z = 3.71 (P = 0.0002)				
**Total (95% CI)**			**100.0%**	**0.70 [0.59, 0.84]**
Heterogeneity: $Tau^2 = 0.00$; $Chi^2 = 4.67$, df = 6 (P = 0.59); $I^2 = 0\%$				
Test for overall effect: Z = 3.80 (P = 0.0001)				
Test for subgroup differences: $Chi^2 = 3.39$, df = 1 (P = 0.07), $I^2 = 70.5\%$				

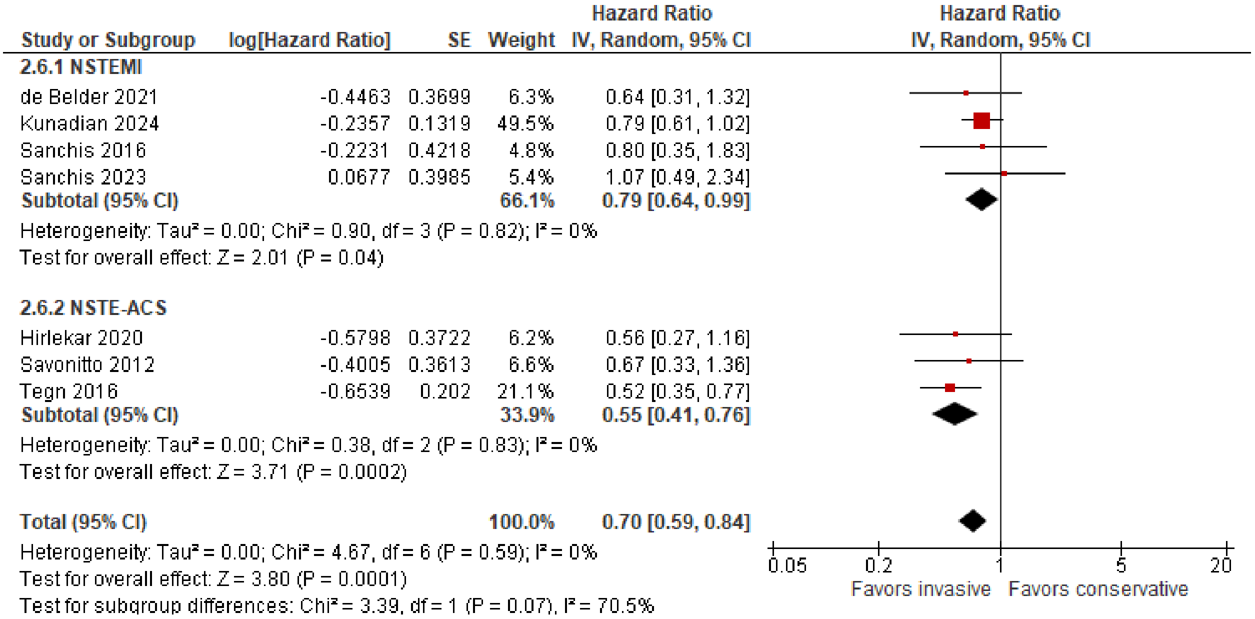

Supplementary Figure S5. Effect of invasive versus conservative management on the risk of cardiovascular mortality in older patients with NSTE-ACS.

	Invasive strategy		Conservative strategy			Risk Ratio
Study or Subgroup	Events	Total	Events	Total	Weight	M-H, Random, 95% CI
de Belder 2021	10	124	10	126	6.7%	1.02 [0.44, 2.36]
Kunadian 2024	119	753	109	765	82.0%	1.11 [0.87, 1.41]
Savonitto 2012	16	154	17	159	11.3%	0.97 [0.51, 1.85]
**Total (95% CI)**		**1031**		**1050**	**100.0%**	**1.09 [0.87, 1.35]**
Total events	145		136			

Heterogeneity: Tau² = 0.00; Chi² = 0.17, df = 2 (P = 0.92); I² = 0%
Test for overall effect: Z = 0.75 (P = 0.45)

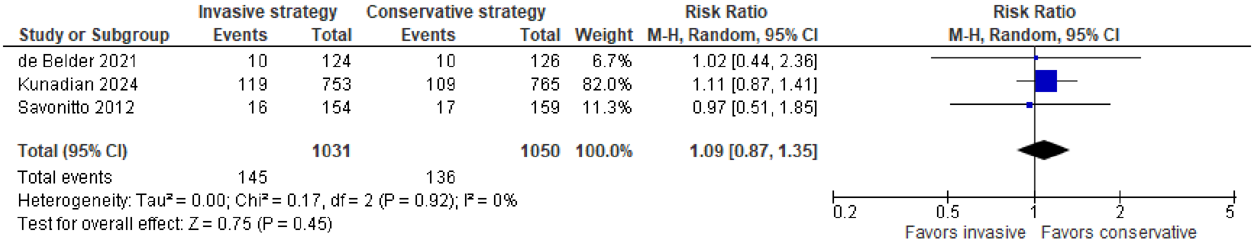

Study or Subgroup	log[Hazard Ratio]	SE	Weight	Hazard Ratio IV, Random, 95% CI
de Belder 2021	0.01	0.4477	7.8%	1.01 [0.42, 2.43]
Kunadian 2024	0.1044	0.1302	92.2%	1.11 [0.86, 1.43]
**Total (95% CI)**			**100.0%**	**1.10 [0.86, 1.41]**

Heterogeneity: Tau² = 0.00; Chi² = 0.04, df = 1 (P = 0.84); I² = 0%
Test for overall effect: Z = 0.78 (P = 0.44)

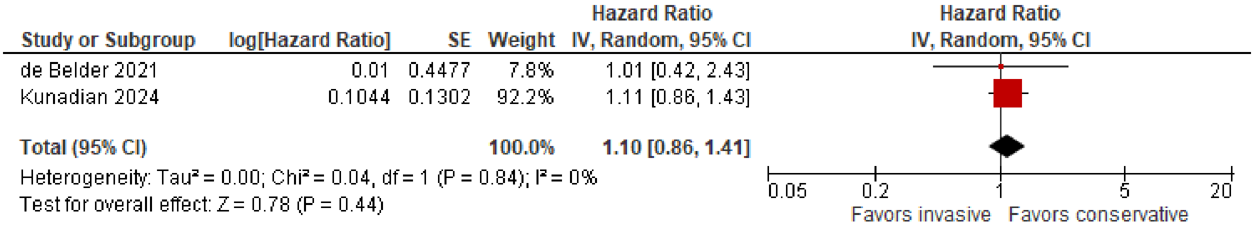

Supplementary Figure S6. Effect of invasive versus conservative management on the risk of revascularization in older patients with NSTE-ACS.

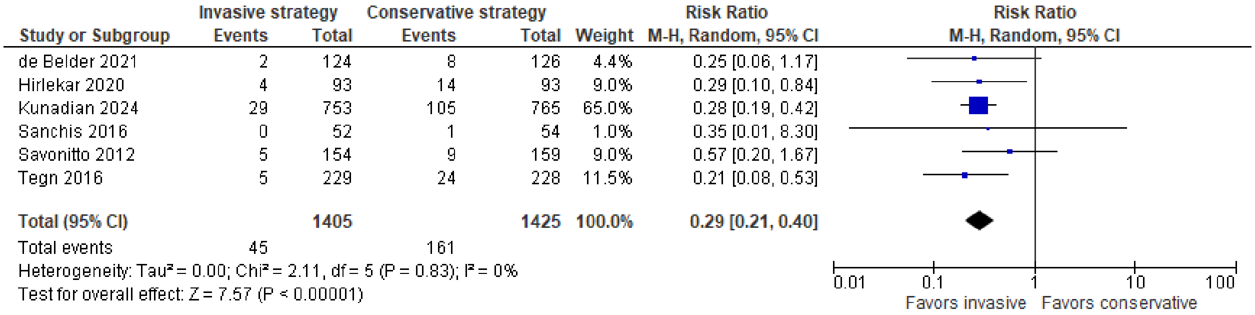

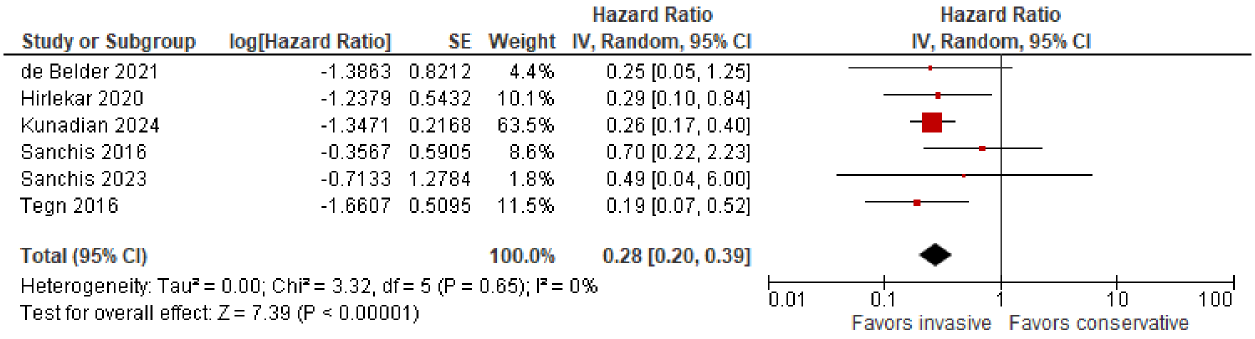

Supplementary Figure S7. Effect of invasive versus conservative management on the risk of stroke in older patients with NSTE-ACS.

Study or Subgroup	Invasive strategy Events	Invasive strategy Total	Conservative strategy Events	Conservative strategy Total	Weight	Risk Ratio M-H, Random, 95% CI
Bach 2004	2	460	5	430	5.1%	0.37 [0.07, 1.92]
de Belder 2021	3	124	3	126	5.5%	1.02 [0.21, 4.94]
Hirlekar 2020	3	93	2	93	4.4%	1.50 [0.26, 8.77]
Kunadian 2024	32	753	40	765	66.5%	0.81 [0.52, 1.28]
Savonitto 2012	0	154	0	159		Not estimable
Tegn 2016	8	229	13	228	18.5%	0.61 [0.26, 1.45]
**Total (95% CI)**		**1813**		**1801**	**100.0%**	**0.77 [0.53, 1.12]**
Total events	48		63			

Heterogeneity: Tau² = 0.00; Chi² = 1.74, df = 4 (P = 0.78); I² = 0%
Test for overall effect: Z = 1.38 (P = 0.17)

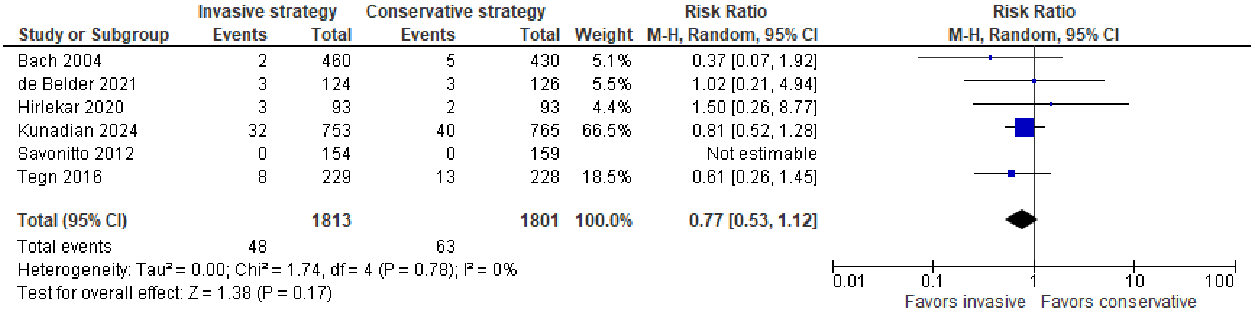

Study or Subgroup	log[Hazard Ratio]	SE	Weight	Hazard Ratio IV, Random, 95% CI
Hirlekar 2020	0.3001	0.903	5.1%	1.35 [0.23, 7.92]
Kunadian 2024	-0.2107	0.236	74.2%	0.81 [0.51, 1.29]
Tegn 2016	-0.5108	0.4467	20.7%	0.60 [0.25, 1.44]
**Total (95% CI)**			**100.0%**	**0.78 [0.52, 1.16]**

Heterogeneity: Tau² = 0.00; Chi² = 0.74, df = 2 (P = 0.69); I² = 0%
Test for overall effect: Z = 1.21 (P = 0.22)

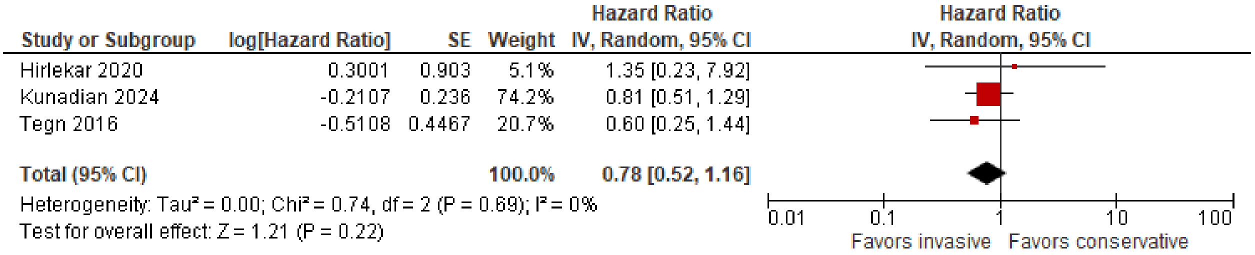

Supplementary Figure S8. Effect of invasive versus conservative management on the risk of severe bleeding in older patients with NSTE-ACS.

Study or Subgroup	Invasive strategy Events	Total	Conservative strategy Events	Total	Weight	Risk Ratio M-H, Random, 95% CI
Bach 2004	52	460	28	430	33.1%	1.74 [1.12, 2.70]
de Belder 2021	7	124	3	126	5.1%	2.37 [0.63, 8.96]
Hirlekar 2020	0	93	0	93		Not estimable
Kunadian 2024	62	753	49	765	42.5%	1.29 [0.90, 1.84]
Sanchis 2016	7	52	10	54	10.8%	0.73 [0.30, 1.77]
Sanchis 2023	8	84	1	83	2.2%	7.90 [1.01, 61.81]
Savonitto 2012	2	154	1	159	1.6%	2.06 [0.19, 22.54]
Tegn 2016	4	229	4	228	4.8%	1.00 [0.25, 3.93]
**Total (95% CI)**		**1949**		**1938**	**100.0%**	**1.43 [1.05, 1.94]**
Total events	142		96			

Heterogeneity: Tau² = 0.02; Chi² = 6.93, df = 6 (P = 0.33); I² = 13%
Test for overall effect: Z = 2.26 (P = 0.02)

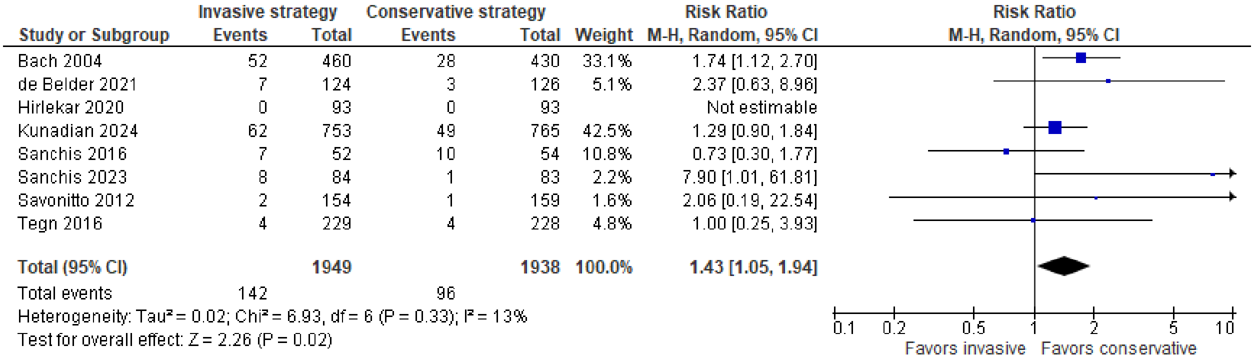

Supplementary Figure S9. Effect of invasive versus conservative management on the risk of major bleeding in older patients with NSTE-ACS.

Study or Subgroup	Invasive strategy Events	Total	Conservative strategy Events	Total	Weight	Risk Ratio M-H, Random, 95% CI
Bach 2004	52	460	28	430	80.2%	1.74 [1.12, 2.70]
de Belder 2021	7	124	3	126	8.8%	2.37 [0.63, 8.96]
Hirlekar 2020	0	93	0	93		Not estimable
Savonitto 2012	2	154	1	159	2.7%	2.06 [0.19, 22.54]
Tegn 2016	4	229	4	228	8.2%	1.00 [0.25, 3.93]
**Total (95% CI)**		**1060**		**1036**	**100.0%**	**1.71 [1.15, 2.54]**
Total events	65		36			

Heterogeneity: Tau² = 0.00; Chi² = 0.86, df = 3 (P = 0.84); I² = 0%
Test for overall effect: Z = 2.67 (P = 0.008)

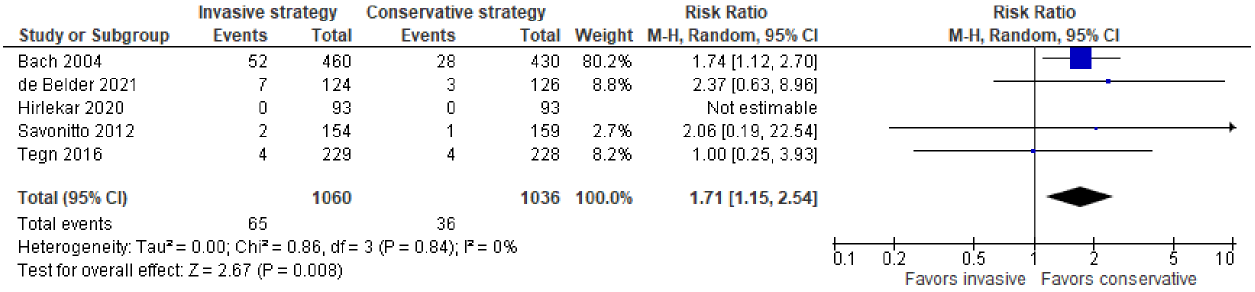

Supplementary Figure S10. Sensitivity analysis by removing Bach 2004 on the effect of invasive versus conservative management on the risk of severe bleeding in older patients with NSTE-ACS.

Study or Subgroup	Invasive strategy Events	Invasive strategy Total	Conservative strategy Events	Conservative strategy Total	Weight	Risk Ratio M-H, Random, 95% CI
Bach 2004	52	460	28	430	0.0%	1.74 [1.12, 2.70]
de Belder 2021	7	124	3	126	8.9%	2.37 [0.63, 8.96]
Hirlekar 2020	0	93	0	93		Not estimable
Kunadian 2024	62	753	49	765	58.0%	1.29 [0.90, 1.84]
Sanchis 2016	7	52	10	54	18.0%	0.73 [0.30, 1.77]
Sanchis 2023	8	84	1	83	3.9%	7.90 [1.01, 61.81]
Savonitto 2012	2	154	1	159	2.9%	2.06 [0.19, 22.54]
Tegn 2016	4	229	4	228	8.4%	1.00 [0.25, 3.93]
**Total (95% CI)**		**1489**		**1508**	**100.0%**	**1.31 [0.86, 1.97]**
Total events	90		68			

Heterogeneity: Tau² = 0.04; Chi² = 5.76, df = 5 (P = 0.33); I² = 13%
Test for overall effect: Z = 1.26 (P = 0.21)

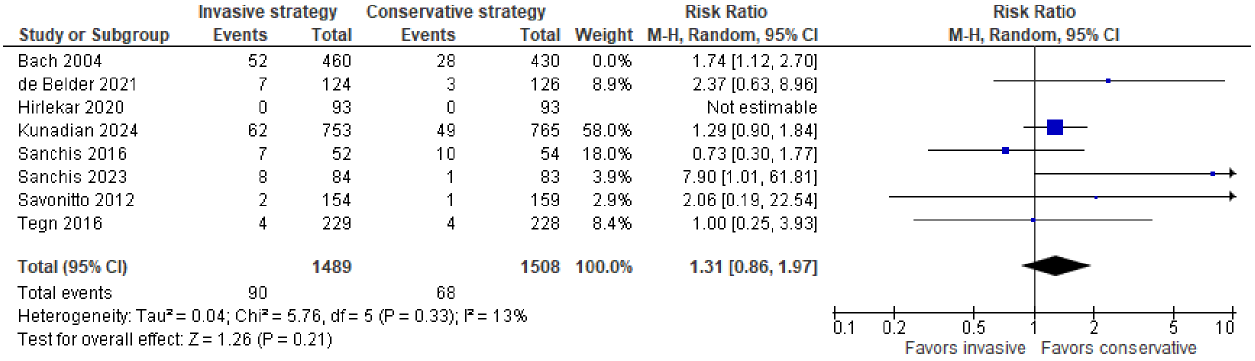

Supplementary Figure S11. Sensitivity analysis by removing Bach 2004 on the effect of invasive versus conservative management on the risk of major bleeding in older patients with NSTE-ACS.

Study or Subgroup	Invasive strategy Events	Invasive strategy Total	Conservative strategy Events	Conservative strategy Total	Weight	Risk Ratio M-H, Random, 95% CI
Bach 2004	52	460	28	430	0.0%	1.74 [1.12, 2.70]
de Belder 2021	7	124	3	126	44.5%	2.37 [0.63, 8.96]
Hirlekar 2020	0	93	0	93		Not estimable
Savonitto 2012	2	154	1	159	13.8%	2.06 [0.19, 22.54]
Tegn 2016	4	229	4	228	41.7%	1.00 [0.25, 3.93]
**Total (95% CI)**		**600**		**606**	**100.0%**	**1.62 [0.67, 3.93]**
Total events	13		8			

Heterogeneity: Tau² = 0.00; Chi² = 0.84, df = 2 (P = 0.66); I² = 0%
Test for overall effect: Z = 1.07 (P = 0.29)

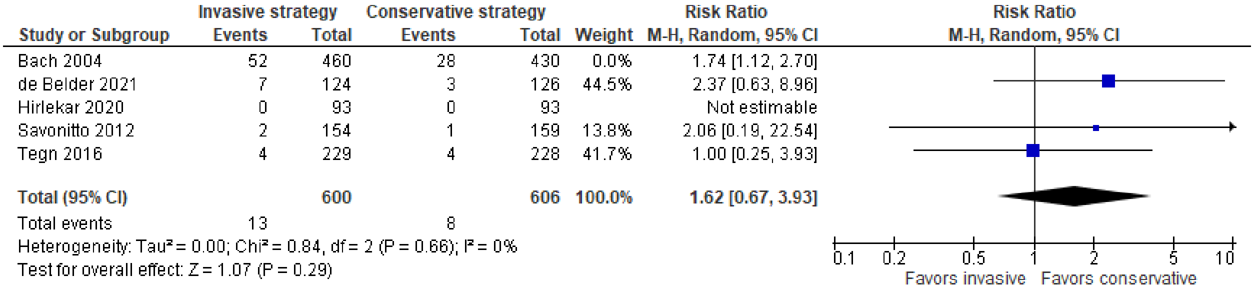

Supplementary Figure S12. Leave-one-out sensitivity analysis of the primary composite endpoint.

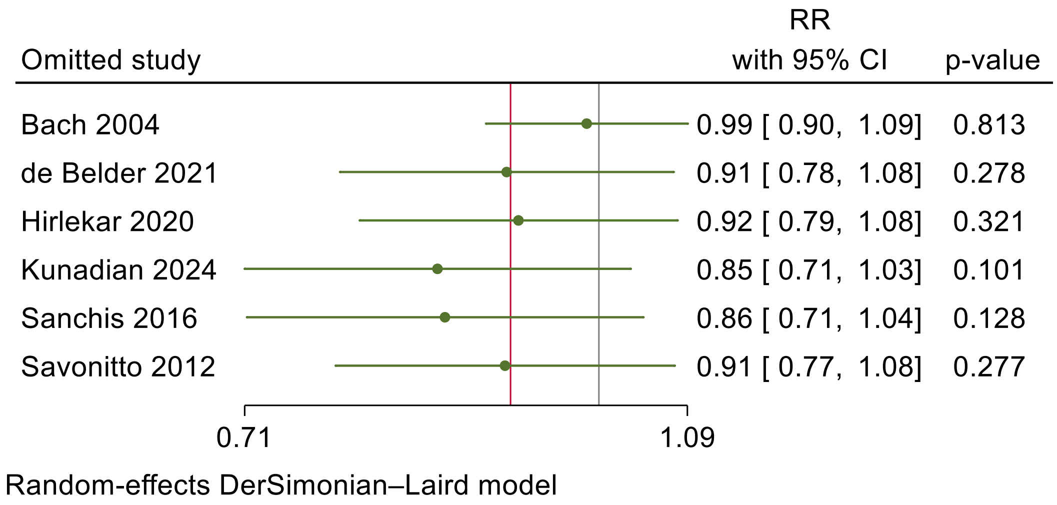

Supplementary Figure S13. Leave-one-out sensitivity analysis of all-cause mortality.

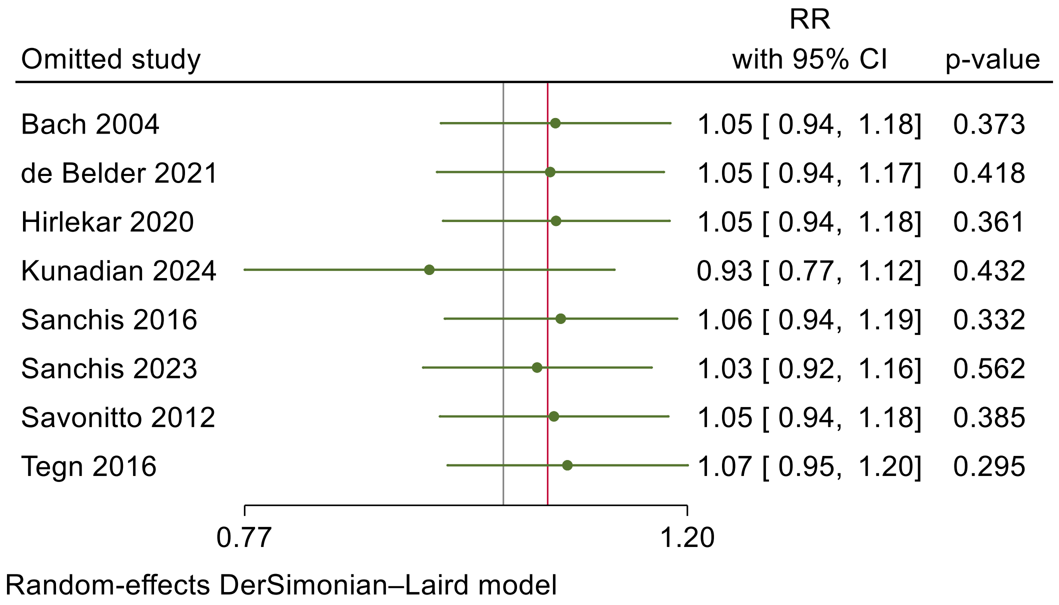

Supplementary Figure S14. Leave-one-out sensitivity analysis of MI.

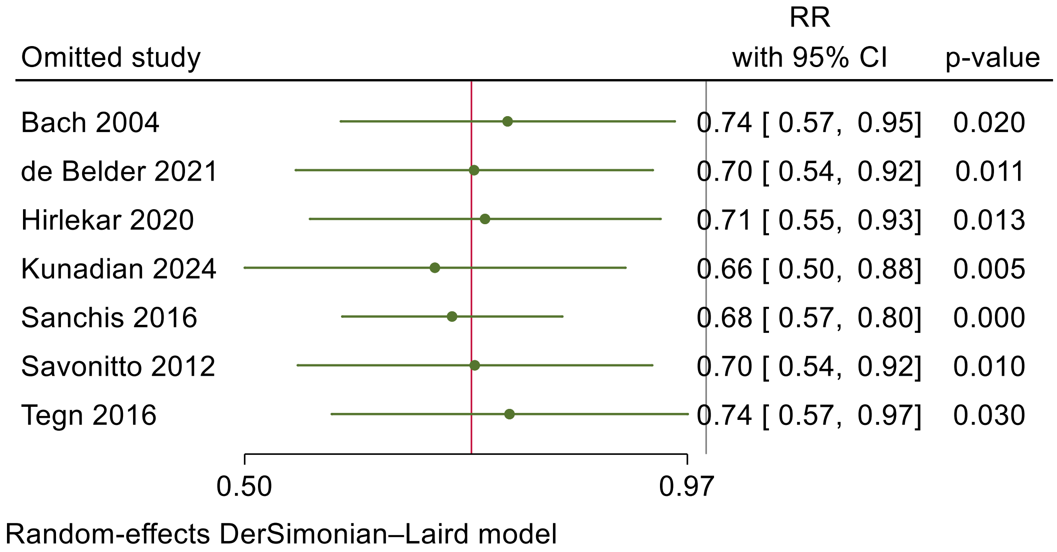

Supplementary Figure S15. Meta-regression of the primary composite endpoint with mean age.

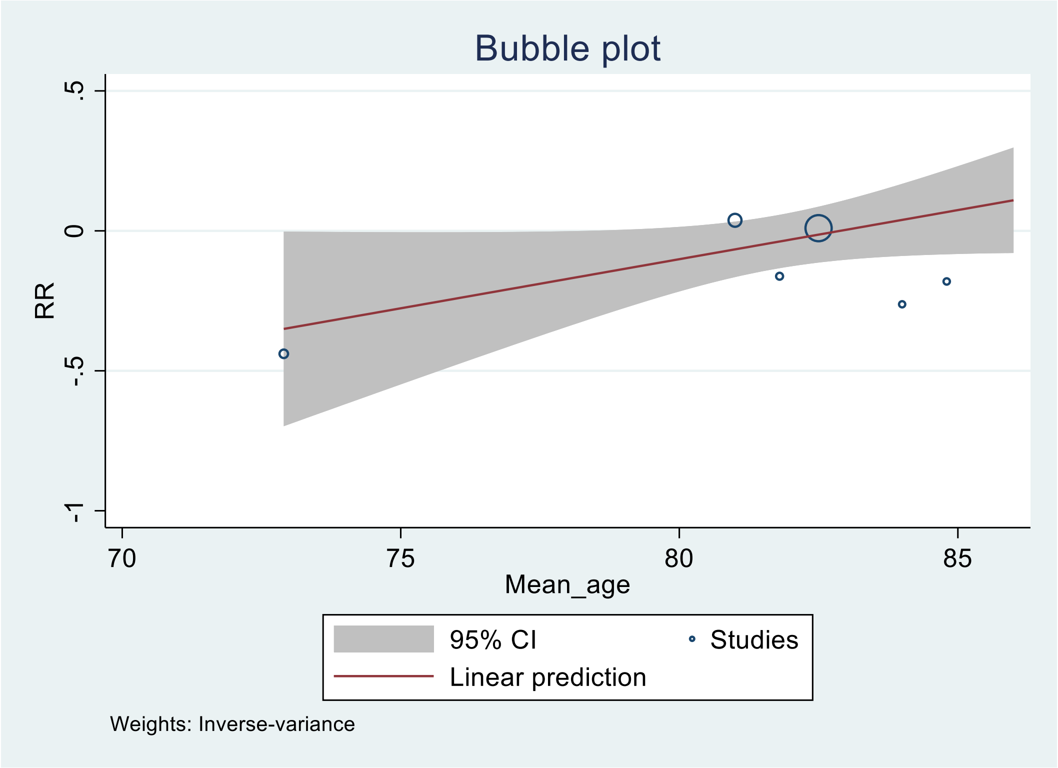


```
  Effect-size label: RR
        Effect size: _meta_es
          Std. err.: _meta_se

Random-effects meta-regression                    Number of obs  =          6
Method: DerSimonian-Laird                         Residual heterogeneity:
                                                              tau2 =          0
                                                            I2 (%) =       0.00
                                                                H2 =       1.00
                                                     R-squared (%) =     100.00
                                                  Wald chi2(1)    =       3.29
                                                  Prob > chi2     =     0.0697
```

_meta_es	Coefficient	Std. err.	z	P>|z|	[95% conf. interval]	
Mean_age	.0350879	.0193474	1.81	0.070	-.0028322	.0730081
_cons	-2.908254	1.581709	-1.84	0.066	-6.008347	.191839

Test of residual homogeneity: Q_res = chi2(4) = 3.72   Prob > Q_res = 0.4446

Supplementary Figure S16. Meta-regression of the primary composite endpoint with male percentage.

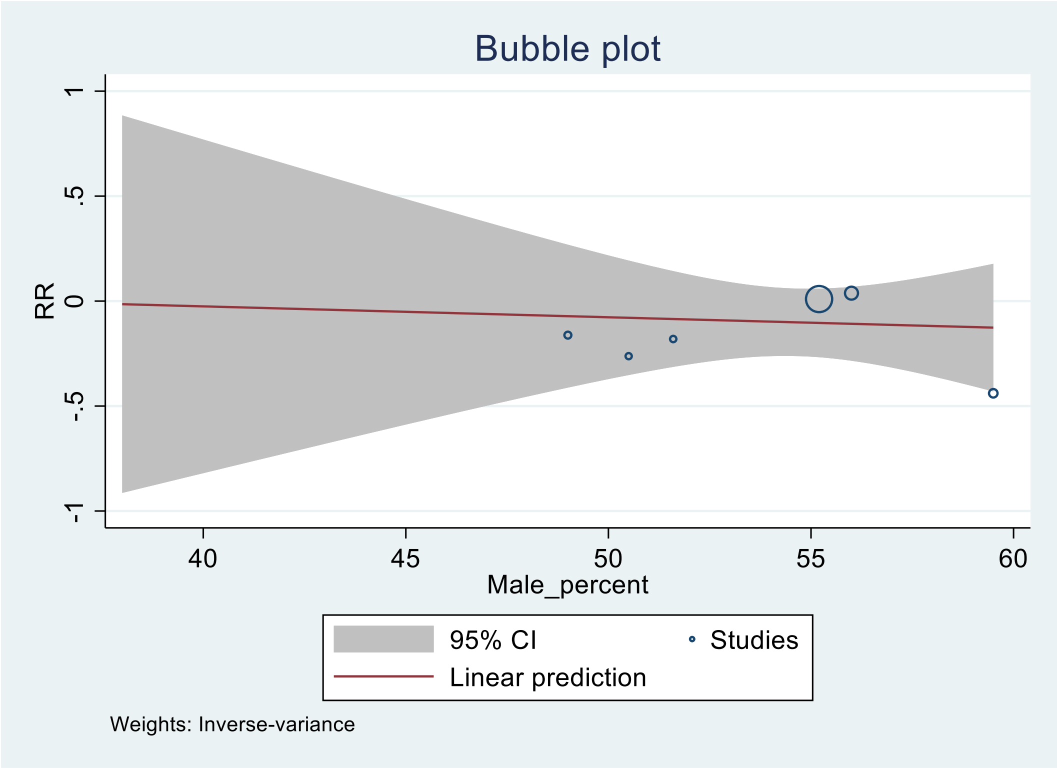


```
Effect-size label: RR
      Effect size: _meta_es
        Std. err.: _meta_se

Random-effects meta-regression                   Number of obs   =          6
Method: DerSimonian-Laird                        Residual heterogeneity:
                                                         tau2 =     .0147
                                                       I2 (%) =     42.73
                                                           H2 =      1.75
                                                R-squared (%) =      0.00
                                                 Wald chi2(1)   =      0.04
                                                 Prob > chi2    =    0.8481
```

_meta_es	Coefficient	Std. err.	z	P>|z|	[95% conf.	interval]
Male_percent	-.0051927	.0271117	-0.19	0.848	-.0583306	.0479451
_cons	.1825532	1.483837	0.12	0.902	-2.725713	3.09082

Test of residual homogeneity: Q_res = chi2(4) = 6.98 Prob > Q_res = 0.1367

Supplementary Figure S17. Meta-regression of the primary composite endpoint with prior MI.

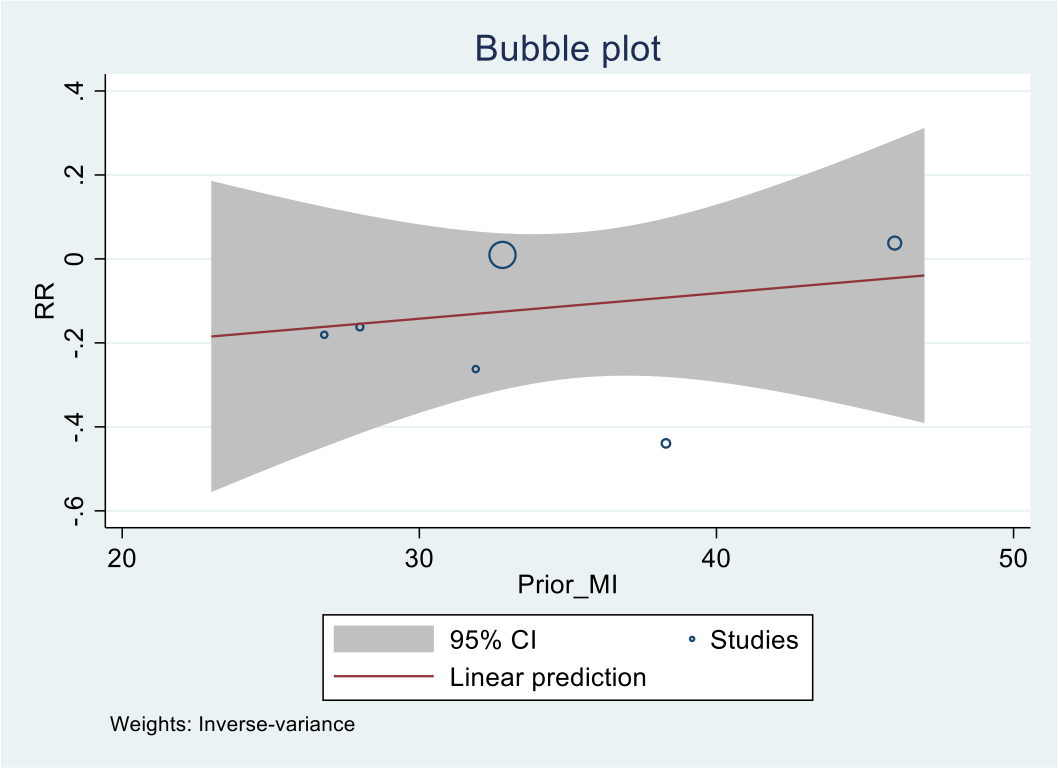


```
. meta regress Prior_MI, random(dlaird)

  Effect-size label: RR
        Effect size: _meta_es
          Std. err.: _meta_se

Random-effects meta-regression                    Number of obs   =          6
Method: DerSimonian-Laird                         Residual heterogeneity:
                                                              tau2 =   .01948
                                                            I2 (%) =    42.16
                                                                H2 =     1.73
                                                     R-squared (%) =     0.00
                                                  Wald chi2(1)    =      0.20
                                                  Prob > chi2     =    0.6529

------------------------------------------------------------------------------
    _meta_es | Coefficient  Std. err.      z    P>|z|     [95% conf. interval]
-------------+----------------------------------------------------------------
    Prior_MI |   .0060546   .0134635     0.45   0.653    -.0203333    .0324425
       _cons |  -.3239754   .4848968    -0.67   0.504    -1.274356    .6264048
------------------------------------------------------------------------------
Test of residual homogeneity: Q_res = chi2(4) =   6.92     Prob > Q_res = 0.1404
```

Supplementary Figure 18. Meta-regression of all-cause mortality with mean age.

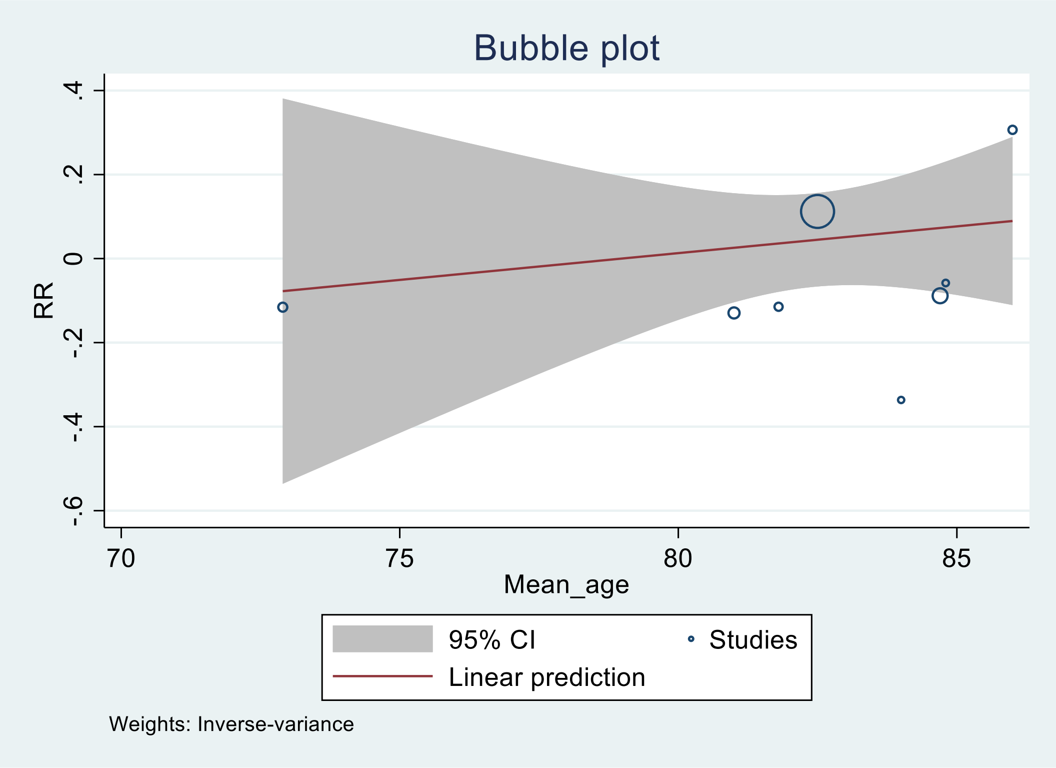


```
  Effect-size label: RR
        Effect size: _meta_es
          Std. err.: _meta_se

Random-effects meta-regression                      Number of obs  =          8
Method: DerSimonian-Laird                           Residual heterogeneity:
                                                                tau2 =        0
                                                              I2 (%) =     0.00
                                                                  H2 =     1.00
                                                       R-squared (%) =     0.00
                                                    Wald chi2(1)   =       0.29
                                                    Prob > chi2    =     0.5923
```

_meta_es	Coefficient	Std. err.	z	P>|z|	[95% conf. interval]	
Mean_age	.0127469	.0238066	0.54	0.592	-.0339133	.059407
_cons	-1.006748	1.963298	-0.51	0.608	-4.854741	2.841246

Test of residual homogeneity: Q_res = chi2(6) = 4.51 Prob > Q_res = 0.6085

Supplementary Figure 19. Meta-regression of all-cause mortality with male percentage.

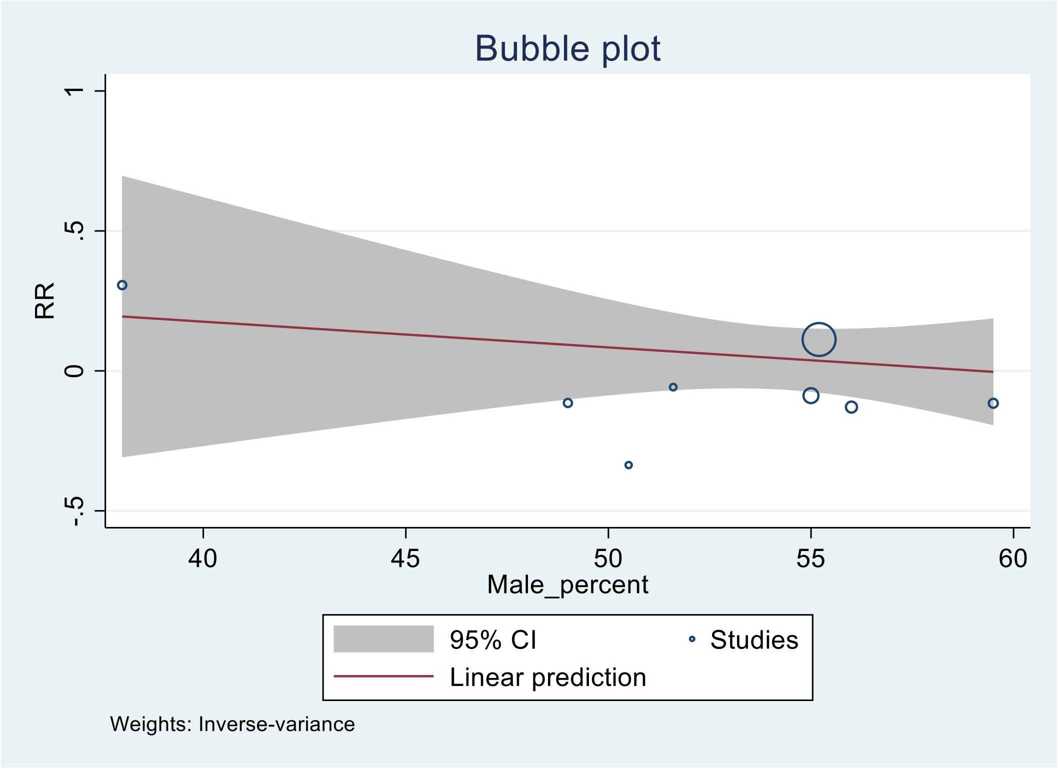


```
  Effect-size label: RR
        Effect size: _meta_es
          Std. err.: _meta_se

Random-effects meta-regression                      Number of obs    =          8
Method: DerSimonian–Laird                           Residual heterogeneity:
                                                                tau2 =          0
                                                              I2 (%) =       0.00
                                                                  H2 =       1.00
                                                      R-squared (%) =       0.00
                                                    Wald chi2(1)     =       0.36
                                                    Prob > chi2      =     0.5481
```

_meta_es	Coefficient	Std. err.	z	P>|z|	[95% conf.	interval]
Male_percent	-.0091904	.0153023	-0.60	0.548	-.0391823	.0208016
_cons	.5434837	.8335446	0.65	0.514	-1.090234	2.177201

Test of residual homogeneity: Q_res = chi2(6) = 4.43   Prob > Q_res = 0.6184

Supplementary Figure 20. Meta-regression of all-cause mortality with prior MI.

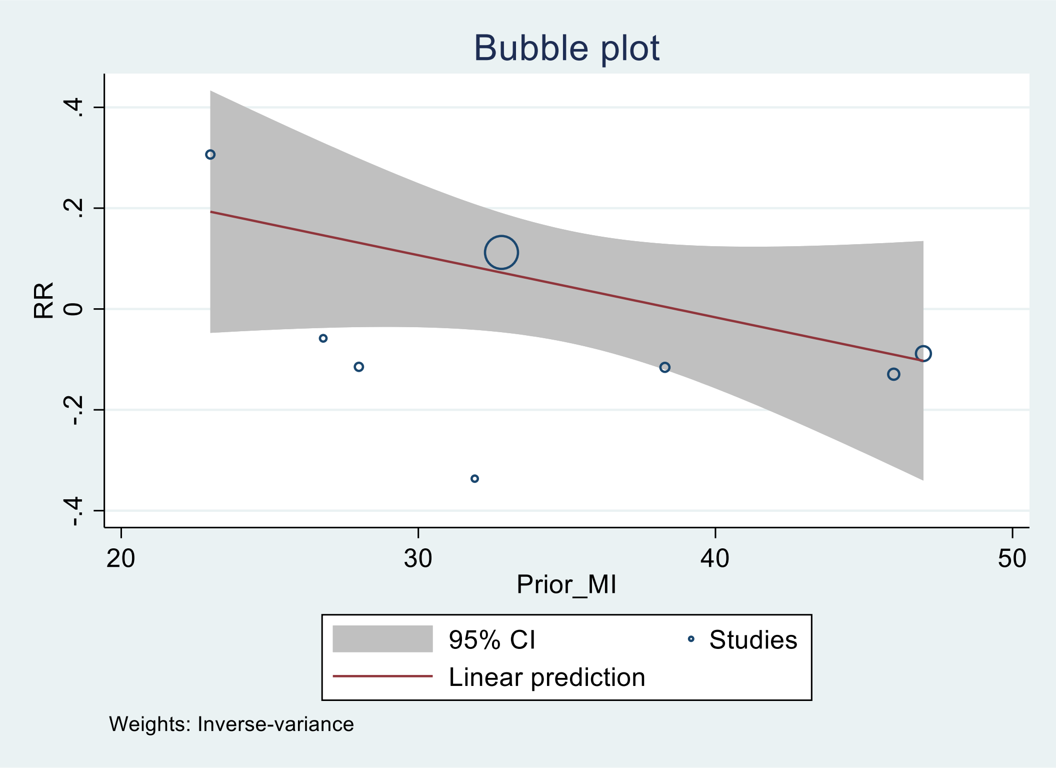


```
  Effect-size label: RR
        Effect size: _meta_es
          Std. err.: _meta_se

Random-effects meta-regression                    Number of obs   =          8
Method: DerSimonian-Laird                         Residual heterogeneity:
                                                              tau2 =         0
                                                            I2 (%) =      0.00
                                                                H2 =      1.00
                                                     R-squared (%) =      0.00
                                                  Wald chi2(1)    =       1.88
                                                  Prob > chi2     =     0.1706
```

_meta_es	Coefficient	Std. err.	z	P>|z|	[95% conf.	interval]
Prior_MI	-.0123241	.0089932	-1.37	0.171	-.0299505	.0053022
_cons	.4764533	.3206139	1.49	0.137	-.1519384	1.104845

```
Test of residual homogeneity: Q_res = chi2(6) =  2.92     Prob > Q_res = 0.8194
```

Supplementary Figure 21. Meta-regression of MI with mean age.

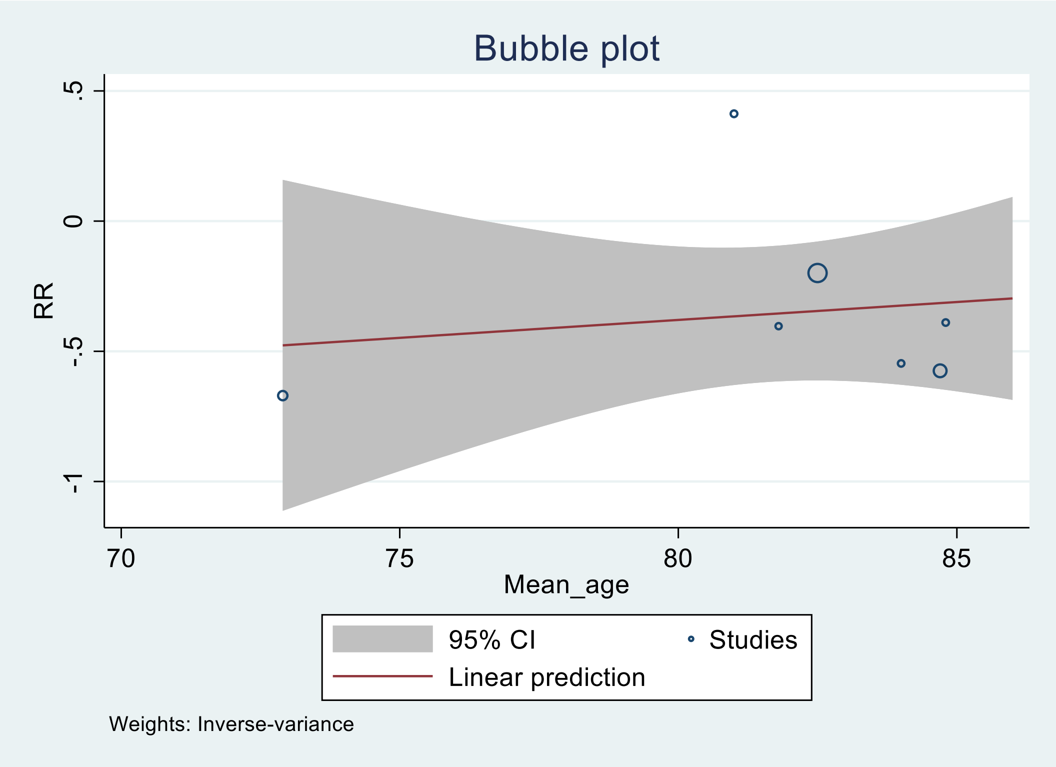


```
Effect-size label: RR
      Effect size: _meta_es
        Std. err.: _meta_se

Random-effects meta-regression                    Number of obs   =          7
Method: DerSimonian–Laird                         Residual heterogeneity:
                                                              tau2 =   .05535
                                                            I2 (%) =    49.17
                                                                H2 =     1.97
                                                     R-squared (%) =     0.00
                                                  Wald chi2(1)    =      0.17
                                                  Prob > chi2     =    0.6845
```

_meta_es	Coefficient	Std. err.	z	P>|z|	[95% conf. interval]	
Mean_age	.0137575	.0338619	0.41	0.685	-.0526106	.0801256
_cons	-1.480039	2.767442	-0.53	0.593	-6.904127	3.944048

Test of residual homogeneity: Q_res = chi2(5) = 9.84 Prob > Q_res = 0.0800

Supplementary Figure 22. Meta-regression of MI with male percentage.

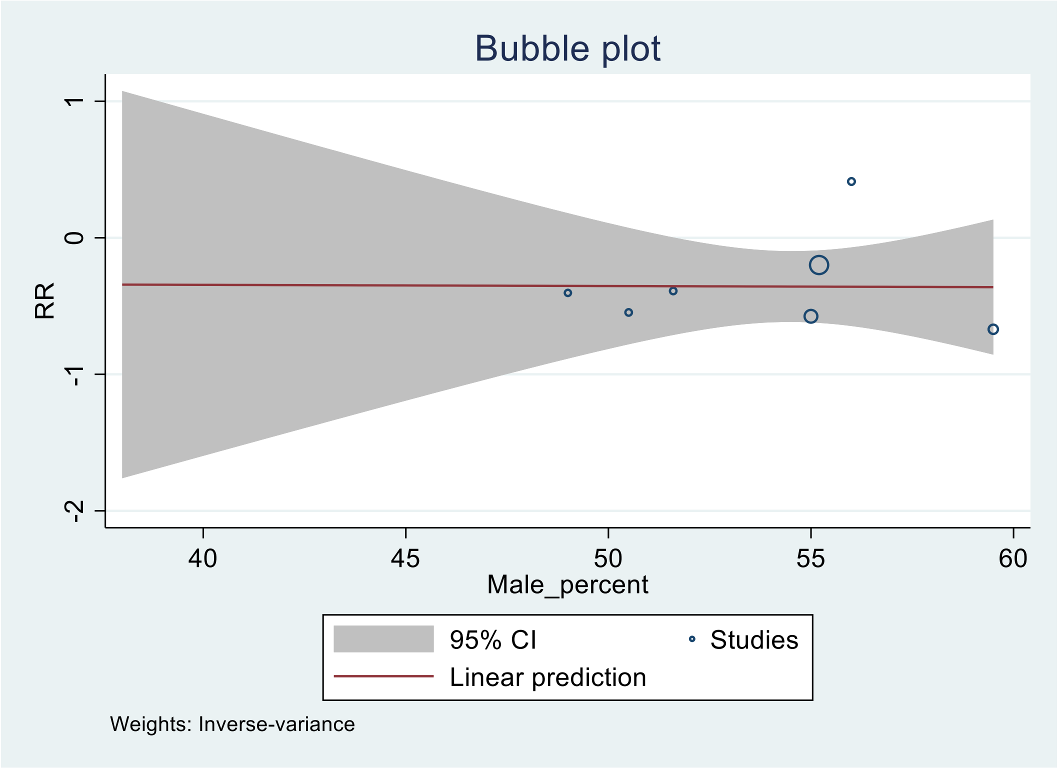


```
  Effect-size label: RR
        Effect size: _meta_es
          Std. err.: _meta_se

Random-effects meta-regression                    Number of obs  =          7
Method: DerSimonian-Laird                         Residual heterogeneity:
                                                              tau2 =   .05435
                                                            I2 (%) =    50.66
                                                                H2 =     2.03
                                                     R-squared (%) =     0.00
                                                  Wald chi2(1)     =     0.00
                                                  Prob > chi2      =   0.9840
```

_meta_es	Coefficient	Std. err.	z	P>|z|	[95% conf.	interval]
Male_percent	-.000863	.0430885	-0.02	0.984	-.0853148	.0835889
_cons	-.3099877	2.352588	-0.13	0.895	-4.920976	4.301001

Test of residual homogeneity: Q_res = chi2(5) = 10.13   Prob > Q_res = 0.0715

Supplementary Figure 23. Meta-regression of MI with prior MI.

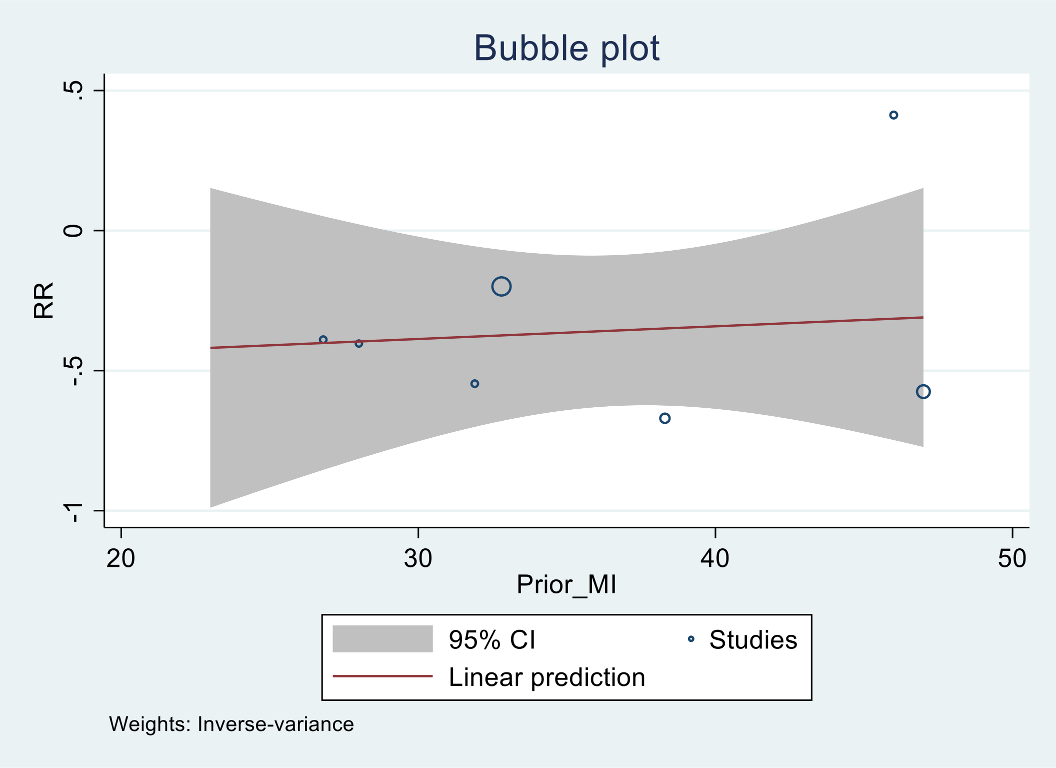


```
  Effect-size label: RR
        Effect size: _meta_es
          Std. err.: _meta_se

Random-effects meta-regression                    Number of obs    =        7
Method: DerSimonian-Laird                         Residual heterogeneity:
                                                              tau2 =   .06256
                                                            I2 (%) =    49.29
                                                                H2 =     1.97
                                                     R-squared (%) =     0.00
                                                  Wald chi2(1)     =     0.06
                                                  Prob > chi2      =   0.8087
```

_meta_es	Coefficient	Std. err.	z	P>|z|	[95% conf.	interval]
Prior_MI	.0045252	.0186884	0.24	0.809	-.0321035	.0411538
_cons	-.5227796	.7001242	-0.75	0.455	-1.894998	.8494386

Test of residual homogeneity: Q_res = chi2(5) = 9.86   Prob > Q_res = 0.0793
